# International consensus conference recommendations on ultrasound education for undergraduate medical students

**DOI:** 10.1186/s13089-022-00279-1

**Published:** 2022-07-27

**Authors:** Richard A. Hoppmann, Jeanette Mladenovic, Lawrence Melniker, Radu Badea, Michael Blaivas, Miguel Montorfano, Alfred Abuhamad, Vicki Noble, Arif Hussain, Gregor Prosen, Tomás Villen, Gabriele Via, Ramon Nogue, Craig Goodmurphy, Marcus Bastos, G. Stephen Nace, Giovanni Volpicelli, Richard J. Wakefield, Steve Wilson, Anjali Bhagra, Jongyeol Kim, David Bahner, Chris Fox, Ruth Riley, Peter Steinmetz, Bret P. Nelson, John Pellerito, Levon N. Nazarian, L. Britt Wilson, Irene W. Y. Ma, David Amponsah, Keith R. Barron, Renee K. Dversdal, Mike Wagner, Anthony J. Dean, David Tierney, James W. Tsung, Paula Nocera, José Pazeli, Rachel Liu, Susanna Price, Luca Neri, Barbara Piccirillo, Adi Osman, Vaughan Lee, Nitha Naqvi, Tomislav Petrovic, Paul Bornemann, Maxime Valois, Jean-Francoise Lanctot, Robert Haddad, Deepak Govil, Laura A. Hurtado, Vi Am Dinh, Robert M. DePhilip, Beatrice Hoffmann, Resa E. Lewiss, Nayana A. Parange, Akira Nishisaki, Stephanie J. Doniger, Paul Dallas, Kevin Bergman, J. Oscar Barahona, Ximena Wortsman, R. Stephen Smith, Craig A. Sisson, James Palma, Mike Mallin, Liju Ahmed, Hassan Mustafa

**Affiliations:** 1 Internal Medicine, University of South Carolina School of Medicine, 6311 Garners Ferry Road, Bldg 3, Room 306, Columbia, SC 29209 USA; 2 Foundation for the Advancement of International Medical Education and Research, Philadelphia, USA; 3 Quality Emergency Department, NewYork-Presbyterian Health System, New York, USA; 4 Internal Medicine and Gastroenterology, Iuliu Hatieganu University of Medicine and Pharmacy, Cluj-Napoca, Romania; 5 Internal Medicine, University of South Carolina School of Medicine, Columbia, USA; 6 Ultrasound and Doppler Department, Hospital de Emergencias “Dr. Clemente Alvarez”, Rosario, Argentina; 7 Eastern Virginia School of Medicine, Norfolk, USA; 8 Emergency Medicine, University Hospitals Cleveland Medical Center, Cleveland, USA; 9 Cardiac Critical Care, King Abdulaziz Medical City, Riyadh, Saudi Arabia; 10 Emergency Medicine, University Medical Centre Maribor, Maribor, Slovenia; 11 Francisco de Vitoria University School of Medicine, Madrid, Spain; 12 Department of Cardiac Anesthesia and Intensive Care, Istituto Cardiocentro Ticino, Ente Ospedaliero Cantonale, Lugano, Switzerland; 13 Emergency Medicine, University of Lleida School of Medicine, Lleida, Spain; 14 Ultrasound Education, Penn State College of Medicine, Hershey, USA; 15 Ultrasound Point of Care, Faculdade de Ciências Médicas e da Saúde de Juiz de Fora - SUPREMA, Juiz de Fora, Brazil; 16 Medical Education and Medicine, University of Tennessee Health Science Center, Memphis, USA; 17 Internal Medicine, Emergency Medicine, San Luigi Gonzaga University Hospital, Turin, Italy; 18 Rheumatology, University of Leeds, Leeds Teaching Hospitals Trust, Leeds, UK; 19 University of South Carolina School of Medicine, Columbia, USA; 20 Internal Medicine, Mayo Clinic, Rochester, USA; 21 Neurology, School of Medicine Texas Tech University Health Sciences Center, Lubbock, USA; 22 Department of Emergency Medicine, The Ohio State University, Columbus, USA; 23 Department Emergency Medicine, University of California Irvine, Irvine, USA; 24 Library Services, University of South Carolina School of Medicine, Columbia, USA; 25 Family Medicine, McGill University, Montreal, Canada; 26 Emergency Medicine, Icahn School of Medicine at Mount Sinai, New York, USA; 27 Radiology and Science Education, Zucker School of Medicine at Hofstra/Northwell Health, Manhasset, USA; 28 Radiology, Sidney Kimmel Medical College, Thomas Jefferson University, Philadelphia, USA; 29 Physiology, University of South Carolina School of Medicine, Columbia, USA; 30 Medicine, Division of General Internal Medicine, University of Calgary, Calgary, Canada; 31 Department of Emergency Medicine, Henry Ford Hospital, Detroit, USA; 32 Department of Internal Medicine, University of South Carolina School of Medicine, Columbia, USA; 33 Internal Medicine, Oregon Health & Science University, Portland, USA; 34 Medicine, University of South Carolina School of Medicine-Greenville, Greenville, USA; 35 Emeritus Department of Emergency Medicine, Perelman University of Pennsylvania School of Medicine, Philadelphia, USA; 36 Internal Medicine, Abbott Northwestern Hospital, Minneapolis, USA; 37 Emergency Medicine and Pediatrics, Icahn School of Medicine at Mount Sinai, New York, USA; 38 Anesthesiologist, Hospital Sírio Libanês, São Paulo, Brazil; 39 Nephology and Critical Care, Barbacena’s School of Medicine, Barbacena, Brazil; 40 Emergency Medicine, Yale School of Medicine, New Haven, USA; 41 Cardiology and Intensive Care, Royal Brompton Hospital, London, England; 42 Emergency and Intensive Care Medicine, King Fahad Specialist Hospital Dammam, Ad Dammām, Saudi Arabia; 43 New York Institute of Technology, Bellmore, USA; 44 Emergency Physician & ED Critical Care, Trauma & Emergency Department, Hospital Raja Permaisuri, Ipoh, Perak Malaysia; 45 Medical Education, University of South Alabama College of Medicine, Mobile, USA; 46 Royal Brompton Hospital Part of Guy’s and St Thomas’ NHS Foundation Trust, London, England; 47 Hôpitaux de Paris, Bobigny, France; 48 Department of Family and Preventive Medicine, University of South Carolina School of Medicine, Columbia, USA; 49 Medicine, McGill and Sherbrooke Universities, Montreal, Canada; 50 Ultrasound Education - Ultrasound Institute, University of South Carolina School of Medicine, Columbia, USA; 51 Critical Care Medicine, Medanta - The Medicity, Gurgaon, India; 52 Universidad de Buenos Aires, Buenos Aires, Argentina; 53 Emergency Medicine and Internal Medicine, Loma Linda University Medical Center, Loma Linda, USA; 54 Emeritus Biomedical Education and Anatomy, The Ohio State University, Columbus, USA; 55 Department of Emergency Medicine, Harvard Medical School, Boston, USA; 56 Emergency Medicine and Radiology, Thomas Jefferson University, Philadelphia, USA; 57 Medical Sonography, University of South Australia Allied Health and Human Performance, Adelaide, Australia; 58 Anesthesia, Critical Care, and Pediatrics, University of Pennsylvania Perelman School of Medicine, Philadelphia, USA; 59 Pediatric Emergency Medicine, Children’s Hospital in Orange California, Orange, USA; 60 Internal Medicine, Virginia Tech Carilion School of Medicine, Roanoke, USA; 61 Family and Community Medicine, University of California - San Francisco, Martinez, USA; 62 Greenwich Ultrasound Services, Greenwich Ultrasound Associates, PC, Greenwich, USA; 63 Department of Dermatology, Faculty of Medicine, Universidad de Chile, Santiago, Chile; 64 Surgery, University of Florida College of Medicine, Gainesville, USA; 65 Emergency Medicine, The University of Texas Health Science Center at San Antonio, San Antonio, USA; 66 Military and Emergency Medicine, F. Edward Hébert School of Medicine, Uniformed Services University of the Health Sciences, Bethesda, USA; 67 Salt Lake City, USA; 68 King Faisal Specialist Hospital and Research Center, Madinah, Kingdom of Saudi Arabia; 69 Internal Medicine, University of Manitoba, Manitoba, Canada

**Keywords:** Ultrasound, Medical student, Education, Undergraduate, International consensus conference, Curriculum recommendations

## Abstract

**Objectives:**

The purpose of this study is to provide expert consensus recommendations to establish a global ultrasound curriculum for undergraduate medical students.

**Methods:**

64 multi-disciplinary ultrasound experts from 16 countries, 50 multi-disciplinary ultrasound consultants, and 21 medical students and residents contributed to these recommendations. A modified Delphi consensus method was used that included a systematic literature search, evaluation of the quality of literature by the GRADE system, and the RAND appropriateness method for panel judgment and consensus decisions. The process included four in-person international discussion sessions and two rounds of online voting.

**Results:**

A total of 332 consensus conference statements in four curricular domains were considered: (1) curricular scope (4 statements), (2) curricular rationale (10 statements), (3) curricular characteristics (14 statements), and (4) curricular content (304 statements). Of these 332 statements, 145 were recommended, 126 were strongly recommended, and 61 were not recommended. Important aspects of an undergraduate ultrasound curriculum identified include curricular integration across the basic and clinical sciences and a competency and entrustable professional activity-based model. The curriculum should form the foundation of a life-long continuum of ultrasound education that prepares students for advanced training and patient care. In addition, the curriculum should complement and support the medical school curriculum as a whole with enhanced understanding of anatomy, physiology, pathophysiological processes and clinical practice without displacing other important undergraduate learning. The content of the curriculum should be appropriate for the medical student level of training, evidence and expert opinion based, and include ongoing collaborative research and development to ensure optimum educational value and patient care.

**Conclusions:**

The international consensus conference has provided the first comprehensive document of recommendations for a basic ultrasound curriculum. The document reflects the opinion of a diverse and representative group of international expert ultrasound practitioners, educators, and learners. These recommendations can standardize undergraduate medical student ultrasound education while serving as a basis for additional research in medical education and the application of ultrasound in clinical practice.

**Supplementary Information:**

The online version contains supplementary material available at 10.1186/s13089-022-00279-1.

## Introduction

The use of ultrasound in medical student (undergraduate) education first appeared in the literature in the 1990s. Early studies from Europe reported enhanced learning of cardiac physiology and human gross anatomy with ultrasound [1, 2]. Since that time, ultrasound as a teaching tool has steadily expanded for both the basic and clinical sciences.

Much of this expansion has been driven by the clinical use of ultrasound at the bedside referred to as point-of-care ultrasound or POCUS. In POCUS, the treating clinician performs ultrasound examinations and interprets the ultrasound images at the bedside to assist with immediate diagnostic and patient management decisions as well as to assist in guiding procedures such as vascular access. The number and diversity of ultrasound clinical applications have grown significantly over the past three decades and ultrasound is now used in almost every practice specialty and subspecialty from primary care to transplant surgery [3, 4].

**Fig. 1:**
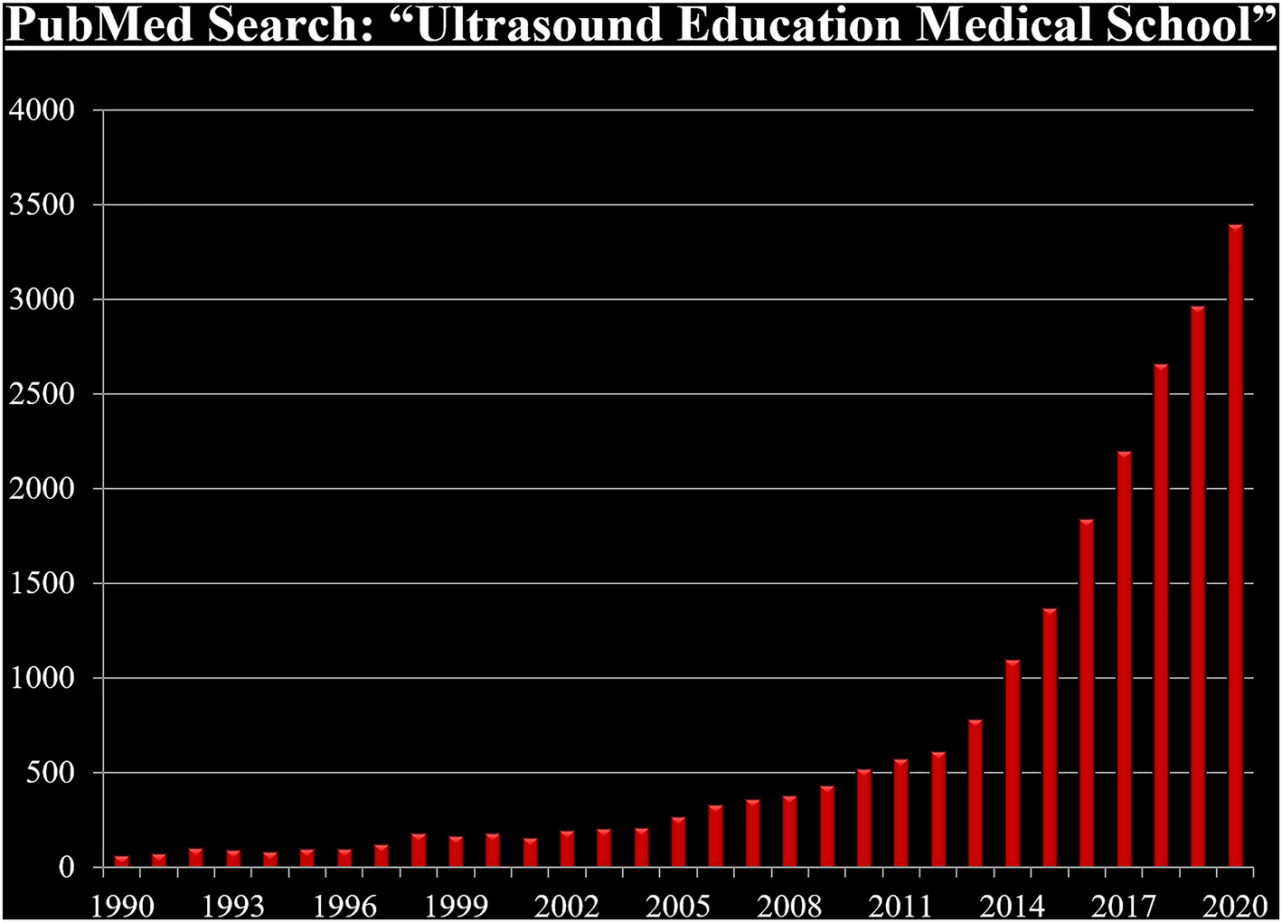
A PubMed search of articles using *ultrasound education medical school* as the query

Most recently, there has been an exponential increase in interest in ultrasound education in medical school as evidenced by the number of ultrasound education-related publications (Fig. 1). Contributing to this rapid rise in interest have been advances in ultrasound technology such as artificial intelligence-assisted image display and automated functions such as computation of the cardiac ejection fraction. These advances have resulted in easier to use hand-held and laptop-sized ultrasound devices with high-quality images. Newer devices are also much more affordable than the previous portable ultrasound machines that initiated the POCUS era. These changes in ease of use, quality of images, functionality, and cost have made teaching large numbers of medical students with ultrasound much more feasible.

Ultrasound education after medical school (postgraduate or residency), outside the traditional ultrasound-use specialties of radiology, cardiology and obstetrics and gynecology, began in the 1990s in the specialties of Emergency Medicine and Critical Care Medicine. Ultrasound leaders in these two specialties have created extensive point-of-care educational resources, have developed postgraduate training competencies and milestones, and have established ultrasound fellowships for advanced training of clinicians, educators, and researchers [5–12]. These contributions have been critical to developing practice standards for the appropriate and safe use of POCUS and the expansion of ultrasound to other specialties and subspecialties.

**Fig. 2:**
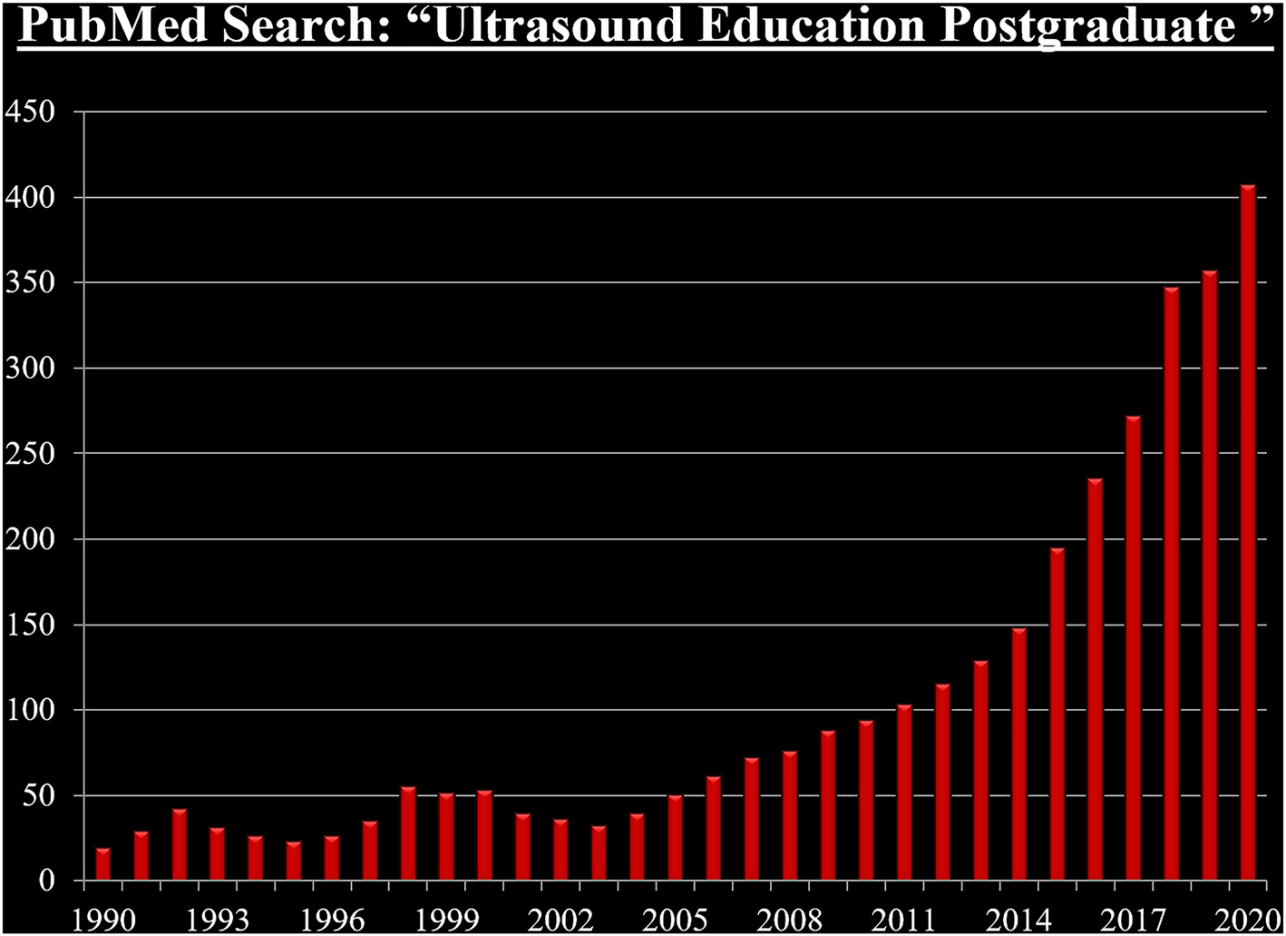
A PubMed search of articles using *ultrasound education postgraduate* as the query

Because of the broad range of ultrasound applications, mounting evidence of the clinical value of point-of-care ultrasound, the availability of educational resources, and the advances in ultrasound technology, many specialties and subspecialties have been incorporating and/or expanding the role of ultrasound in their postgraduate training programs [13–17]. Postgraduate ultrasound education publications like those in undergraduate education are showing an exponential rise, as depicted in Fig. 2.

Thus, a continuum of ultrasound education is evolving, beginning in undergraduate medical education. Necessary and central to the success of such an educational continuum will be the establishment of foundational ultrasound knowledge, attitude, and skills for the definition of basic ultrasound competencies with attendant milestones and assessment. To this end, the Society of Ultrasound in Medical Education (SUSME) and the World Interactive Network Focused on Critical Ultrasound (WINFOCUS) conducted this international conference to provide consensus recommendations for developing a global ultrasound curriculum for undergraduate medical education. Such recommendations will serve as the basis for establishing ultrasound as a core clinical competency for all medical school graduates and prepare these graduates for future advanced clinical training.

Four domains of statements related to medical student ultrasound education were addressed: the scope of an international consensus ultrasound curriculum, the rationale for the curriculum, the characteristics of the curriculum, and curricular content. This last domain was of particular importance as the lack of standardized content for ultrasound education has been a significant obstacle to the broad adoption of ultrasound in medical student education [18–20]. Such standardization is necessary to facilitate faculty development as well as promote ultrasound educational and clinical research to further develop evidence that guides the use of ultrasound in medical education and clinical practice [21–23].

A modified Delphi consensus method was used that included a systematic search of the literature, the GRADE method of assessment of level of quality of evidence, and RAND appropriateness methodology for the degree of consensus and strength of recommendations. Even though the number of publications on ultrasound education is relatively large, those of high-quality evidence-based studies are still quite limited. Thus, it was anticipated that this international consensus conference would need to rely heavily on expert opinion in establishing the most appropriate ultrasound content for medical student education. A large diverse group of expert ultrasound practitioners, researchers, and educators was recruited to participate in the process to enhance the validity of the consensus and ensure the best recommendations were achieved.

Overall, the consensus process involved expert voting panelists and expert consultants, along with the education stakeholders of medical students and residents. This broad group of participants was designed to capture consensus recommendations applicable across educational settings with variable curricular structures, needs, and resources, as well as to address several limitations of previous papers on ultrasound curricular content for medical students. These prior publications were usually limited by medical specialty or discipline representation and the breadth of their institutional applicability and accreditation standards. Recommendations on methods of teaching ultrasound and student assessment were beyond the scope of this consensus conference.

## Methods

### Literature search

Initial PubMed literature searches were conducted in 2016 and 2017 using the following query: *((("medical students"[TIAB] OR "medical education"[TIAB]) OR "education, medical"[MeSH Terms]) OR "students, medical"[MeSH Terms]) AND ((("ultrasonics"[MeSH Terms] OR "ultrasonography"[MeSH Terms]) OR "ultrasound"[TIAB]) OR "ultrasonography"[TIAB] OR "ultrasonics"[TIAB]) AND (("1997/01/01"[PDat]: "3000/12/31"[PDat])).*

The search resulted in the identification of 1832 records. These records were then limited to English only and 20 years resulting in 1556 records. These records were then reviewed in duplicate by two steering committee members with inclusion and exclusion criteria to identify all records relative to medical student ultrasound education resulting in 275 records.

In addition to the primary PubMed searches, secondary parallel searches were performed in the following databases: Academic Search (68 records), CINAHL (58 records), Cochrane Library (3 records), ERIC (6 records), PsychINFO (11 records), and Web of Science (544 records).

**Fig. 3:**
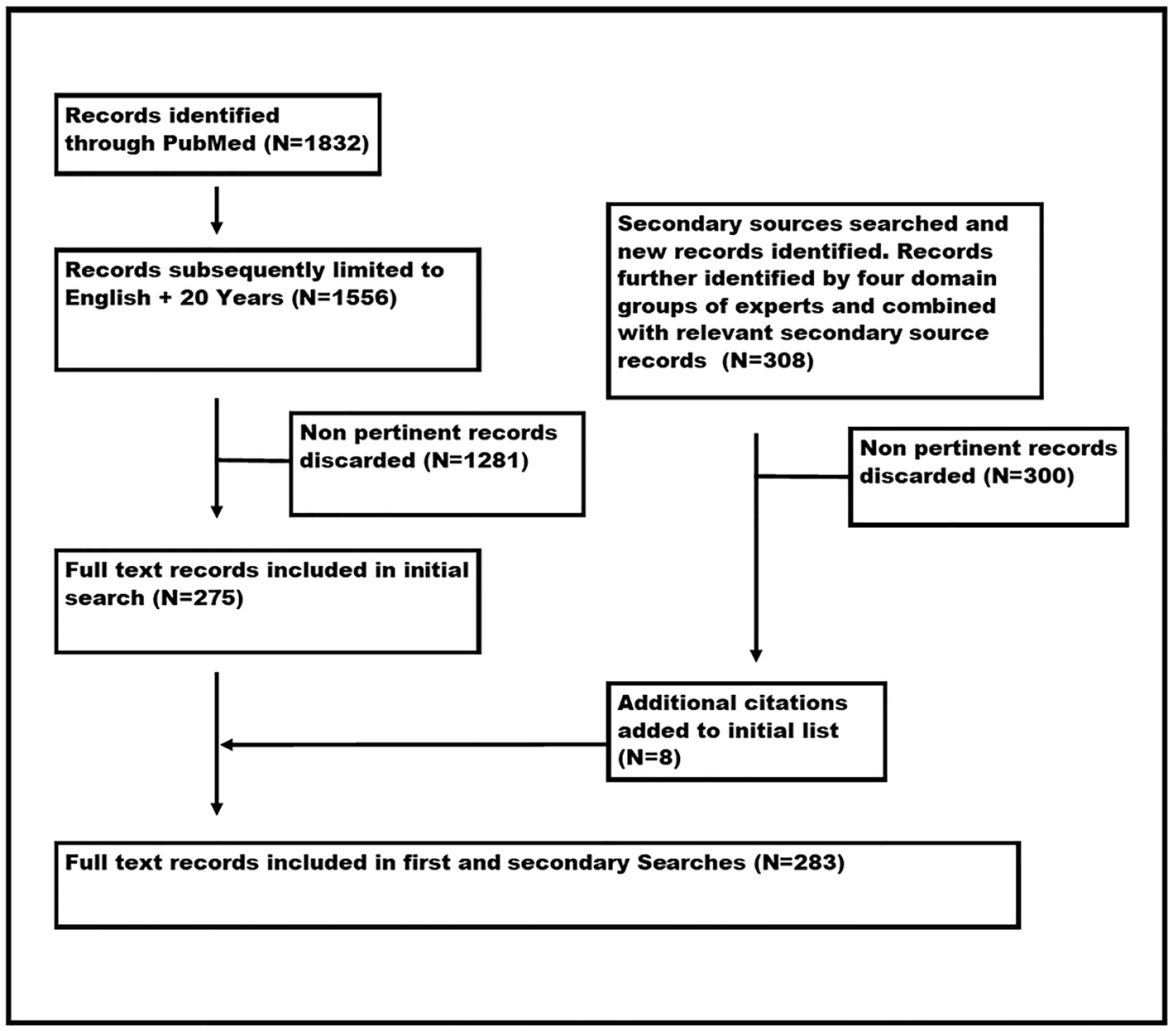
Literature search for relevant records

The records from the secondary searches were compared to the initial PubMed record list and duplications were removed. These records were screened for relevancy and added to records recommended by the Domain leaders from literature searched through 2018. A total of 283 records were used for the consensus process as shown in Fig. 3. Search results were made available to all consensus conference participants on a central *International Consensus Conference on Ultrasound in Medical Education* website with other consensus resources such as published ultrasound standards and guidelines, community forums, updated searches, and links to other relevant sites. The website remained active throughout the entire consensus conference process.

### Consensus conference steering committee, domains, and domain task teams

A consensus conference steering committee of eight members knowledgeable in ultrasound, education, and consensus processes was formed to guide the consensus process. Four of the members represented the Society of Ultrasound in Medical Education (SUSME) and four represented the World Interactive Network Focused on Critical Ultrasound (WINFOCUS). Six different specialties and subspecialties from four countries were represented on the steering committee. One member of the steering committee had advanced training in epidemiology with expertise in consensus methodology and oversaw the methodology of the process. The steering committee agreed on four general topics or domains of ultrasound in medical education to develop essential statements and consensus recommendations. These included Domain 1: Scope of the International Consensus Curriculum; Domain 2: Rationale for the Curriculum; Domain 3: Characteristics of the Curriculum; and Domain 4: Curricular Content.

Four domain task teams were formed with co-chairs to further evaluate the subject matter within their domain, identify additional literature, and develop relevant PICO consensus statements (population, intervention, comparison of intervention, outcomes) for Domains 1–3 and curricular content items for Domain 4. Discussion and editing of the domains among panelists and consultants took place at four international meetings on ultrasound (World Congress on Ultrasound in Medical Education in Lubbock, TX, September 2016, World Congress on Ultrasound in Medical Education in Montreal, Canada, October 2017, WINFOCUS World Congress on Ultrasound in Emergency and Critical Care in Dubai, United Arab Emirates, February 2019, and World Congress on Ultrasound in Medical Education in Irvine, CA, September 2019).

The individual curricular content items of Domain 4 were initially determined by a task team of eight expert ultrasound practitioners and educators with input from steering committee members. Content items were further discussed during the international ultrasound meetings. These content items were identified from the ultrasound literature and published ultrasound guidelines, but in general lacked evidence documenting their educational value per se. Thus, the content list was created to suggest content that could be of value to medical student education; the final recommendations on content were made by the expert voting panel consensus.

### Voting panelists, consultants, and medical student/resident stakeholders

Nationally and internationally recognized clinicians, basic scientists, educators and researchers were invited to participate in the consensus process either as voting panelists or consultants based on their area of ultrasound expertise, clinical experience, educational experience, record of publications, and leadership positions in professional societies, hospital systems, and/or academia. All voting panelists and consultants completed a professional profile form and submitted a curriculum vitae and a declaration of interest/conflict of interest form for consideration.

#### Voting panelists

Voting panelists were selected to provide representation across clinical specialties and subspecialties, basic science expertise, geographic distribution, educational experience, and familiarity with the various medical education systems throughout the world. Broad yet balanced representation was sought to strengthen the validity of the consensus process for an integrated ultrasound curriculum that would span the basic and clinical sciences of medical student education and prepare medical students to pursue postgraduate medical education training in any specialty or subspecialty they should choose and within a variety of global medical educational systems.

As summarized in Table 1, the voting panelists represented a diverse group of educators and practitioners with a wide range of areas of expertise and experience, including over 20 medical and surgical specialties, adult and pediatric expertise, two foundational basic science disciplines, and non-physician ultrasound practitioners. Sixteen nations were represented (Table 2). When possible, voting panelists within the same specialty were chosen to cover a spectrum of ultrasound interests or primary foci in an attempt to balance areas of ultrasound expertise (i.e., radiology—general, vascular, musculoskeletal, dermatology).

**Table 1:** Basic science and clinical specialties and subspecialties represented on the international consensus conference for medical education voting panel

Specialty	Subspecialty	Number of panelists
Anatomy		3
Anesthesiology		3
Cardiology	Adult	1
	Pediatrics	1
Critical Care	Adult	5
	Pediatrics	1
Emergency Medicine	Adult	21
	Pediatrics	1
Family Medicine		3
Hospitalist		3
Intensive Care		4
Internal Medicine	General	6
	Gastroenterology	2
	Hematology	1
	Nephrology	3
	Pulmonary	1
	Rheumatology	2
Neurology		1
Obstetrics/gynecology	General	2
	Maternal fetal medicine	1
Pediatrics		3
Physician assistant	Emergency medicine	1
Physiology		1
Radiology	General	1
	Vascular	1
	Musculoskeletal	1
	Dermatology	1
	Pediatrics	1
Sonographer	OB/GYN and general	1
	Cardiac and vascular	2
Surgery	General	2
	Trauma	2
	Critical care	1

**Table 2:** Countries represented on the voting panel

1	Argentina
2	Australia
3	Brazil
4	Canada
5	Chile
6	France
7	India
8	Italy
9	Malaysia
10	Saudi Arabia
11	Slovenia
12	Spain
13	Switzerland
14	Romania
15	United Kingdom
16	United States of America

The total number of panelist specialties, subspecialties, and area of special expertise exceeds the total individual panelists number of 64 since a number of panelists were formally trained, practiced, and taught in more than one area of ultrasound.

As a group, voting panelists accounted for many contributions to the ultrasound literature. Panelists had published an average of 32.7 peer-reviewed publications and 7.1 ultrasound book chapters. Thirty-one panelists had served as an ultrasound book editor. All panelists had been involved in national and international ultrasound societies and 86.6% had held leadership positions in these organizations.

Over 90% of panelists had greater than 5 years of experience in teaching medical students (93.7%), postgraduate residents (94.6%) and practicing physicians (96.4%). Many panelists had greater than 15 years of ultrasound teaching experience and had served as ultrasound education directors in their academic institutions and/or clinical ultrasound training program (83.3%). Eighty percent had greater than 5 years of experience teaching other healthcare providers such as nurses, nurse practitioners, physician assistants, and midwives. Eighty-eight percent of the panelists had been involved in ultrasound research for greater than 5 years.

#### Consultants

The credentials of the consultants were very similar to those of the voting panelists. The consultants were involved in discussions of the statements and recommendations. The consultants participated in a preliminary voting survey of Round 1 statements and recommendations. The results of the consultants’ survey were then made available to the voting panelists for consideration. Fifty consultants participated in the preliminary Round 1 voting. The decision to include participants as voting panelists or consultants was made on the time individuals had to commit to the process, the need for representative balance in specialty, subspecialty, and science discipline as well as level of expertise and geographical representation.

#### Medical students and residents

Medical students and residents as stakeholders in their education were given opportunities to provide input through online communities (Disqus) and complete a preliminary Round 1 survey. The results of this survey were made available to the voting panelists for consideration. Medical students and residents who participated were identified through various global medical student ultrasound interest groups and chief resident listings. No systematic attempt was made to seek a balanced representation of students and residents. Therefore, their input should be considered as that of a sample based on interest and convenience. Twenty-one students and residents participated in the preliminary voting and their responses were pooled together.

### Voting and evaluations of recommendations

A modified Delphi method was used for two rounds of voting. The level of quality of evidence was determined by the GRADE method and the RAND appropriateness method was used for the degree of consensus and strength of recommendations [24, 25]. Voting was done anonymously. Levels of quality of evidence for literature supporting a statement were rated as: Level 1 (high), Level 2A (moderate), Level 2B (low), Level 3 (very low). A nine-point Likert scale of appropriateness for each PICO statement was used with 1–3 denoting inappropriate, 4–6 denoting somewhat appropriate, and 7–9 denoting appropriate. Using RAND Rules to determine whether a statement was recommended, strongly recommended, or not recommended included an assessment of the median level of appropriateness, the degree of consensus, and the percentage of disagreement of the voters.

During the consensus process, voting was accomplished using online customized forms. Voters were sent the voting link and allowed approximately 2 weeks to complete the surveys. Reminders were sent during the open voting period. Participants could complete the survey at one time or could return to complete the survey as convenient for them.

In April 2019, a voting survey of statements of all four domains was distributed to all consultants with explicit voting instructions. Fifty of 54 consultants completed the survey (93%). Summary graphic and numerical results of consultants’ responses were made available to the voting panelists via an active link for consideration during the voting period.

In June 2019, a voting survey of statements of all four domains was distributed to students and residents. A total of 21 responded. Summary graphic and numerical results of student /resident responses were made available to the voting panel via an active link for consideration during the voting period.

In August 2019, Round 1 of the voting survey of statements of all four domains was distributed to all 64 voting panelists and 64 completed the survey (100%). For Domains 1–3, the number of supporting references at each Grade of Evidence for each statement was listed with the statement. Available to all voting panelists at the time of voting was access via electronic links to results of the consultants’ survey responses and the students’/residents’ survey responses for each statement and each curricular content item. In addition, a comprehensive PDF of all domain statements with comments, rationales, and supporting citations with links to abstracts and/or original articles or documents was also available. Links were also available of descriptions and explanations of the RAND Rules and the GRADE process and scoring.

In September 2019, Round 2 of the voting survey was conducted with the voting panelists. Fifty-nine of 64 panelists completed the survey (92%). Twenty-five new curricular content elements were added based on Round 1 panelists’ comments and discussion and feedback at the consensus conference meeting held in Irvine, CA, during the World Congress on Ultrasound in Medical education between Round 1 and Round 2.

During this second round of voting, statistics for each statement, level of consensus (perfect, very good, good, some, and no consensus) as well as individual panelists’ Round 1 comments and relevant comments from the Irvine consensus conference meeting were made available for panelists to consider prior to voting.

## Voting results

Table 3 lists all statements in Domains 1–3 with references that were considered as evidence for each statement, the median appropriateness score for Round 2, the degree of consensus, the level of evidence, and the strength of the recommendation. Table 4 lists all Domain 4 content items considered for an undergraduate medical student ultrasound curriculum.

**Table 3:** Domains 1–3 voting results

Code	Statement	Refs.	Median voting score	Degree of consensus	Level of quality of evidence	Strength of recommendation
Domain 1: scope of consensus conference curriculum						
D1.1	The ICC will produce consensus recommendations on “An integrated ultrasound curriculum” (“curriculum”) for undergraduate medical education (medical school)	[26–38]	8.38	VGC	Level 1 = 0, Level 2A = 0, Level 2B = 2, Level 3 = 11	Strongly recommend
D1.2	The curriculum forms the foundation for ultrasound as a core clinical competency for all graduates regardless of specialty choice	[3, 13, 27, 33–55]	8.45	VGC	Level 1 = 0, Level 2A = 3, Level 2B = 7, Level 3 = 14	Strongly recommend
D1.3	The curriculum provides the foundation of ultrasound for all medical students, regardless of where their medical degree is obtained or the specific designation of their degree	[27, 28, 56–58]	8.07	GC	Level 1 = 0, Level 2A = 0, Level 2B = 0, Level 3 = 5	Recommend
D1.4	The curriculum can serve as a valuable resource for the development of ultrasound training programs for non-physician healthcare providers such as advanced nurse practitioners and physician assistants	[27, 42, 59–66]	7.62	GC	Level 1 = 0, Level 2A = 0, Level 2B = 4, Level 3 = 3	Recommend
Domain 2: rationale for curriculum						
D2.1	The curriculum prepares students for POCUS (point-of-care ultrasound use) in future clinical work	[35, 37, 38, 67–73]	8.36	GC	Level 1 = 0, Level 2A = 0, Level 2B = 7, Level 3 = 3	Recommend
D2.2	The curriculum facilitates teaching of fundamental sciences	[74–81]	8.09	GC	Level 1 = 0, Level 2A = 1, Level 2B = 2, Level 3 = 3	Recommend
D2.3	The curriculum enhances the learning of clinical sciences	[82, 83]	8.16	GC	Level 1 = 0, Level 2A = 0, Level 2B = 1, Level 3 = 1	Recommend
D2.4	The curriculum facilitates integration of fundamental sciences	[82, 84–87]	7.95	GC	Level 1 = 0, Level 2A = 0, Level 2B = 1, Level 3 = 4	Recommend
D2.5	The curriculum enhances physical examination skills	[44, 45, 80, 86, 88–99]	8.24	GC	Level 1 = 0, Level 2A = 2, Level 2B = 8, Level 3 = 1	Recommend
D2.6	The curriculum enhances clinical problem solving	[73, 100, 101]	8.16	SC	Level 1 = 0, Level 2A = 1, Level 2B = 2, Level 3 = 0	Recommend
D2.7	The curriculum prepares learners for additional clinical training and/or practice opportunities	[69, 73, 102–115]	8.34	GC	Level 1 = 0, Level 2A = 1, Level 2B = 9, Level 3 = 5	Recommend
D2.8	The curriculum enhances the overall educational experience	[33, 35, 70, 82, 84, 116–121]	8.47	VGC	Level 1 = 0, Level 2A = 0, Level 2B = 2, Level 3 = 9	Strongly recommend
D2.9	Medical students can learn basic ultrasound	[33, 35, 70–72, 75, 122–143]	8.64	VGC	Level 1 = 0, Level 2A = 0, Level 2B = 13, Level 3 = 7	Strongly recommend
D2.10	Medical students can learn ultrasound-guided procedures	[122, 143–151]	8.16	GC	Level 1 = 0, Level 2A = 2, Level 2B = 4, Level 3 = 3	Recommend
Domain 3: characteristics of the curriculum						
D3.1	The ultrasound curriculum forms the foundation for ultrasound training along a continuum of medical education from undergraduate through graduate to continuing medical education	[18, 19, 33, 37, 38, 135, 152–155]	8.60	VGC	Level 1 = 0, Level 2A = 1, Level 2B = 2, Level 3 = 7	Strongly recommend
D3.2	The ultrasound curriculum supports undergraduate medical education	[18, 19, 106, 107, 135, 152–154]	8.29	GC	Level 1 = 0, Level 2A = 0, Level 2B = 2, Level 3 = 0	Recommend
D3.3	The ultrasound curriculum prepares learners for future additional clinical training and/or practice opportunities	[18, 19, 106, 107, 135, 152–154]	8.14	GC	Level 1 = 0, Level 2A = 0, Level 2B = 2, Level 3 = 0	Recommend
D3.4	The ultrasound curriculum is developed in accordance with accepted standards for medical education as defined by national and international accrediting bodies	[27, 36, 106, 156–158]	7.76	GC	Level 1 = 0, Level 2A = 0, Level 2B = 1, Level 3 = 1	Recommend
D3.5	The ultrasound curriculum lends itself to a competency-based model that includes measurable outcomes and markers of progression toward those outcomes (milestones)	[38, 81, 155, 159, 160]	7.90	GC	Level 1 = 0, Level 2A = 0, Level 2B = 0, Level 3 = 1	Recommend
D3.6	The ultrasound curriculum can incorporate ultrasound knowledge, skills, attitudes, and professional judgment into entrustable professional activities (EPAs) as appropriate for patient care	[38, 81, 155, 159, 160]	7.98	GC	Level 1 = 0, Level 2A = 0, Level 2B = 0, Level 3 = 1	Recommend
D3.7	The ultrasound curriculum enhances the learning of fundamental sciences that are relevant to the understanding of human pathophysiology and the practice of medicine	[19, 116, 118, 158, 161, 162]	8.24	GC	Level 1 = 0, Level 2A = 0, Level 2B = 1, Level 3 = 3	Recommend
D3.8	The ultrasound curriculum enhances the learning of clinical sciences through the integration of ultrasound into clinical problem solving	[3, 44, 45, 99, 100, 154, 163]	8.34	VGC	Level 1 = 0, Level 2A = 1, Level 2B = 4, Level 3 = 0	Strongly recommend
D3.9	The ultrasound curriculum enhances the learning of clinical sciences through the care of patients at their point of care	[3, 44, 45, 99, 100, 154, 163]	8.17	GC	Level 1 = 0, Level 2A = 1, Level 2B = 4, Level 3 = 0	Recommend
D3.10	The ultrasound curriculum includes opportunities for self-directed learning and assessment	[19, 31, 155]	7.83	GC	Level 1 = 0, Level 2A = 0, Level 2B = 0, Level 3 = 0	Recommend
D3.11	The ultrasound curriculum encourages life-long learning	[19, 31, 155]	7.62	GC	Level 1 = 0, Level 2A = 0, Level 2B = 0, Level 3 = 0	Recommend
D3.12	The ultrasound curriculum is based on evidence and expert opinion	[5, 106, 164–172]	8.28	VGC	Level 1 = 0, Level 2A = 2, Level 2B = 0, Level 3 = 8	Strongly recommend
D3.13	The ultrasound curriculum is consistent with recommendations and guidelines of well-established specialty organizations	[5, 106, 164–172]	8.28	VGC	Level 1 = 0, Level 2A = 2, Level 2B = 0, Level 3 = 8	Strongly recommend
D3.14	The ultrasound curriculum is consistent with recommendations and guidelines of regulatory bodies with significant experience in ultrasound	[5, 106, 164–172]	8.12	VGC	Level 1 = 0, Level 2A = 2, Level 2B = 0, Level 3 = 8	Strongly recommend

From left to right: Code of domain statement, Statement, Reference numbers of relevant literature, Median appropriateness score of Round 2, Degree of consensus. (VGC = very good consensus, GC = good consensus, SC = some consensus, NC = no consensus), Level of quality of evidence (Level 1 = high, Level 2A = moderate, Level 2B = low, Level 3 = very low, strength of recommendation (strongly recommend, recommend, not recommend). The table includes article Refs. [26–172]

**Table 4:** List of all curricular content items in Domains 4 with the median appropriateness score for Round 2, the degree of consensus, and the strength of the recommendation

Domain 4 and curricular content items	Median voting score	Degree of consensus	Strength of recommendation
Part I. Basic foundations of POCUS			
Students should be familiar with the following terms as they are related to the basic physics of ultrasound			
D4.1 Wavelength	8.43	VGC	Strongly recommend
D4.2 Amplitude	8.21	VGC	Strongly recommend
D4.3 Frequency	8.71	VGC	Strongly recommend
D4.4 Attenuation	8.66	VGC	Strongly recommend
D4.5 Refraction	8.48	VGC	Strongly recommend
D4.6 Absorption	8.29	VGC	Strongly recommend
D4.7 Scatter	8.41	VGC	Strongly recommend
D4.8 Transmission	8.52	VGC	Strongly recommend
D4.9 Resolution	8.74	VGC	Strongly recommend
D4.10 Reflection	8.53	VGC	Strongly recommend
D4.11 Aliasing	7.72	SC	Recommend
Students should be able to explain the fundamental principles of ultrasound for the following modes			
D4.12 B mode	8.78	VGC	Strongly recommend
D4.13 M Mode	8.75	VGC	Strongly recommend
D4.14 Color Flow	8.50	VGC	Strongly recommend
D4.15 Power Doppler	7.16	SC	Recommend
D4.16 Spectral Doppler	7.03	SC	Recommend
Students should demonstrate an understanding of the components and parts of ultrasound probes			
D4.17 Housing/body	8.31	VGC	Strongly recommend
D4.18 Piezoelectric crystals	8.23	VGC	Strongly recommend
D4.19 Marker/indicator	8.88	VGC	Strongly recommend
D4.20 Cord	8.57	VGC	Strongly recommend
Students should know the indications for and limitations of each of the following probes			
D4.21 Linear	8.81	VGC	Strongly recommend
D4.22 Curved array	8.79	VGC	Strongly recommend
D4.23 Phased array	8.81	VGC	Strongly recommend
D4.24 Endocavity	8.10	GC	Recommend
Students should demonstrate: - appropriate storage of probes - appropriate care of the probes - cleaning and disinfection of the probes			
D4.24 Probe storage	8.78	VGC	Strongly recommend
D4.26 Probe care	8.88	VGC	Strongly recommend
D4.27 Cleaning/disinfection	8.88	VGC	Strongly recommend
Students should utilize the following transducer manipulations:			
D4.28 Slide	8.63	VGC	Strongly recommend
D4.29 Rock	8.48	VGC	Strongly recommend
D4.30 Sweep	7.97	SC	Recommend
D4.31 Fan	8.40	SC	Recommend
D4.32 Pressure/compression	8.55	GC	Recommend
D4.33 Rotation	8.64	GC	Recommend
Students should be familiar with the following image descriptions			
D4.34 In plane and out of plane	8.79	VGC	Strongly recommend
D4.35 Deep and superficial	8.86	VGC	Strongly recommend
D4.36 Medial and lateral	8.81	GC	Recommend
D4.37 Cranial and caudal	8.74	GC	Recommend
D4.38 Coronal	8.79	VGC	Strongly recommend
D4.39 Sagittal	8.79	VGC	Strongly recommend
D4.40 Transverse	8.84	VGC	Strongly recommend
Students should have the ability to discuss sonographic characteristics of tissues			
D4.41 Anechoic	8.84	VGC	Strongly recommend
D4.42 Hyperechoic	8.84	VGC	Strongly recommend
D4.43 Hypoechoic	8.78	GC	Recommend
D4.44 Isoechoic	8.76	GC	Recommend
D4.45 Mixed echogenicity	8.62	VGC	Strongly recommend
D4.46 Homogeneous	8.74	VGC	Strongly recommend
D4.47 Heterogeneous	8.74	VGC	Strongly recommend
D4.48 Solid	8.72	VGC	Strongly recommend
D4.49 Cystic	8.78	VGC	Strongly recommend
Students should demonstrate the ability to optimize an ultrasound image by utilizing the following machine adjustments			
D4.50 Presets	8.52	VGC	Strongly recommend
D4.51 Gain	8.81	VGC	Strongly recommend
D4.52 Time-gain Compensation	8.36	SC	Recommend
D4.53 Frequency	8.33	SC	Recommend
D4.54 Depth	8.84	VGC	Strongly recommend
D4.55 Focal point	8.22	SC	Recommend
D4.56 Probe marker	8.89	VGC	Strongly recommend
Students should recognize basic ultrasound artifacts used in clinical diagnosis and explain the cause of each			
D4.57 Reverberation (A and B lines)	8.72	VGC	Strongly recommend
D4.58 Comet tail	8.40	GC	Recommend
D4.59 Posterior acoustic shadowing	8.67	VGC	Strongly recommend
Students should understand the following additional basic common artifacts			
D4.60 Air artifact	8.67	VGC	Strongly recommend
D4.61 Mirroring	8.58	VGC	Strongly recommend
D4.62 Acoustic enhancement	8.66	VGC	Strongly recommend
D4.63 Acoustic shadowing	8.69	VGC	Strongly recommend
D4.64 Mirror image	8.57	VGC	Strongly recommend
D4.65 Twinkle	7.45	SC	Recommend
Students should describe the indications for each of the following			
D4.66 Brightness B mode	8.71	VGC	Strongly recommend
D4.67 Motion M mode	8.71	VGC	Strongly recommend
D4.68 Doppler flow	8.52	VGC	Strongly recommend
D4.69 Power Doppler	7.46	SC	Recommend
D4.70 Spectral Doppler	7.33	SC	Recommend
Students should be able to acquire images with			
D4.71 Brightness B mode (gray scale)	8.57	VGC	Strongly recommend
D4.72 Motion M mode	8.41	VGC	Strongly recommend
D4.73 Color Doppler	7.96	SC	Recommend
D4.74 Power Doppler	6.66	NC	Not recommend
D4.75 Spectral Doppler	6.14	NC	Not recommend
Students should be able to identify the following basic tissues by ultrasound			
D4.76 Fluid	8.81	VGC	Strongly recommend
D4.77 Fat	8.14	GC	Recommend
D4.78 Soft tissue	8.66	VGC	Strongly recommend
D4.79 Bone	8.78	VGC	Strongly recommend
D4.80 Muscle	8.60	GC	Recommend
D4.81 Cartilage	7.76	SC	Recommend
D4.82 Tendon	8.16	SC	Recommend
D4.83 Nerve	8.17	SC	Recommend
D4.84 Blood vessels	8.79	GC	Recommend
Students, when examining a patient with ultrasound, should demonstrate proper care for the patient through			
D4.85 Professional communication regarding use of ultrasound	8.86	VGC	Strongly recommend
D4.86 Obtaining informed consent	8.64	VGC	Strongly recommend
D4.87 Respect for patient privacy	8.88	VGC	Strongly recommend
D4.88 Respect for patient comfort	8.88	VGC	Strongly recommend
D4.89 Appropriate positioning of the patient	8.84	VGC	Strongly recommend
D4.90 Completion of documentation of findings	8.59	VGC	Strongly recommend
D4.91 An understanding of the principle of ALARA (As Low As Reasonably Achievable)	8.45	VGC	Strongly recommend
D4.92 Students should correlate ultrasound images with clinical findings	8.95	VGC	Strongly recommend
Part II specific views, structures, and pathology			
Heart and vessels			
Views			
D4.93 Parasternal long axis	8.80	VGC	Strongly recommend
D4.94 Parasternal short axis	8.61	GC	Recommend
D4.95 Apical four chamber	8.54	GC	Recommend
D4.96 Subxiphoid (subcostal)	8.66	VGC	Strongly recommend
D4.97 IVC Transverse	8.20	SC	Recommend
D4.98 IVC Longitudinal	8.57	GC	Recommend
Structures and physiology			
D4.99 Left atrium, right atrium, left ventricle, right ventricle	8.77	VGC	Strongly recommend
D4.100 Mitral value	8.61	VGC	Strongly recommend
D4.101 Aortic valve	8.60	VGC	Strongly recommend
D4.102 Tricuspid valve	8.39	GC	Recommend
D4.103 Pulmonic valve	6.58	NC	Not recommend
D4.104 Myocardium	8.65	VGC	Strongly recommend
D4.105 Pericardium	8.68	VGC	Strongly recommend
D4.106 Descending aorta	8.39	GC	Recommend
D4.107 Aortic arch	7.11	SC	Recommend
D4.108 Abdominal aorta	8.63	VGC	Strongly recommend
D4.109 Aortic bifurcation into common iliac arteries	8.34	GC	Recommend
D4.110 Renal arteries	6.3	NC	Not recommend
D4.111 Dorsalis Pedis	6.88	NC	Not recommend
D4.112 Posterior Tibialis	6.81	NC	Not recommend
D4.113 Correlation of sonographic cardiac cycle with EKG	7.96	SC	Recommend
D4.114 Carotid arteries, including common carotid	8.26	VGC	Strongly recommend
D4.115 Inferior Vena Cava	8.61	VGC	Strongly recommend
D4.116 IVC size	8.33	VGC	Strongly recommend
D4.117 IVC respiratory variations	8.37	VGC	Strongly recommend
D4.118 Internal jugular vein	8.56	VGC	Strongly recommend
Clinical pathology			
D4.119 Poor contractility	8.67	VGC	Strongly recommend
D4.120 LVEF less than 40%	7.95	GC	Recommend
D4.121 LVEF greater than 40%	7.82	GC	Recommend
D4.122 Enlarged chamber size	8.36	VGC	Strongly recommend
D4.123 Enlarged left atrium	8.05	SC	Recommend
D4.124 Enlarged left ventricle	8.18	SC	Recommend
D4.125 Enlarged right Ventricle	8.11	SC	Recommend
D4.126 Distinguish between arterial versus venous flow on Doppler	7.93	SC	Recommend
D4.127 Ventricular Septal Defect	5.39	NC	Not recommend
D4.128 Patent Foramen Ovale	4.98	NC	Not recommend
D4.129 Presence of pericardial effusion	8.72	VGC	Strongly recommend
D4.130 Distinguish between pleural effusion and pericardial effusion	8.63	GC	Recommend
D4.131 Left Ventricular Hypertrophy	7.51	SC	Recommend
D4.132 Idiopathic hypertrophic subaortic stenosis	5.89	NC	Not recommend
D4.133 Right ventricular strain from PE	7.34	SC	Recommend
D4.134 Size of abdominal aortic aneurysm	8.35	GC	Recommend
D4.135 Abdominal dissection of an aortic aneurysm	7.07	NC	Not recommend
D4.136 Decreased vascular volume by IVC collapsibility	8.09	SC	Recommend
D4.137 Lower extremity deep venous thrombosis (DVT)	7.91	SC	Recommend
D4.138 Upper extremity deep venous thrombosis (DVT)	6.86	NC	Not recommend
D4.139 Carotid plaques	6.75	NC	Not recommend
D4.140 Carotid stenosis	6.39	NC	Not recommend
Lungs and chest			
Views			
D4.141 Anterior chest bilaterally	8.60	VGC	Strongly recommend
D4.142 Lateral and posterior chest	8.44	VGC	Strongly recommend
D4.143 Longitudinal across two ribs	8.47	VGC	Strongly recommend
D4.144 Costophrenic angles bilaterally	8.54	VGC	Strongly recommend
Structures and physiology			
D4.145 Visceral pleura	8.21	GC	Recommend
D4.146 Parietal pleura	8.24	GC	Recommend
D4.147 Lung sliding	8.77	VGC	Strongly recommend
D4.148 A lines—A Profile	8.75	VGC	Strongly recommend
Clinical pathology			
D4.149 B lines—B Profile	8.60	VGC	Strongly recommend
D4.150 Pneumothorax—absence of pleural sliding	8.16	GC	Recommend
D4.151 Lung point	8.37	GC	Recommend
D4.152 Pulmonary edema	8.20	GC	Recommend
D4.153 Pleural effusion	8.79	VGC	Strongly recommend
D4.154 Presence of consolidation	8.39	GC	Recommend
D4.155 Sliding curtain sign	7.51	GC	Recommend
D4.156 Acute respiratory distress syndrome (ARDS)	6.77	SC	Recommend
Abdomen			
Views			
D4.157 Epigastric	8.32	VGC	Strongly recommend
D4.158 Left upper quadrant	8.68	VGC	Strongly recommend
D4.159 Right upper quadrant	8.74	VGC	Strongly recommend
D4.160 Lower abdomen	8.51	VGC	Strongly recommend
Structures and physiology			
D4.161 Liver	8.74	VGC	Strongly recommend
D4.162 Size	7.75	SC	Recommend
D4.163 Parenchyma	8.09	GC	Recommend
D4.164 Portal vein	7.98	SC	Recommend
D4.165 Hepatic vein	8.00	SC	Recommend
D4.166 Gallbladder	8.54	VGC	Strongly recommend
D4.167 Stomach	7.35	SC	Recommend
D4.168 Pancreas	6.91	NC	Not recommend
D4.169 Right and left kidneys	8.77	VGC	Strongly recommend
D4.170 Size	8.11	GC	Recommend
D4.171 Cortex	8.07	GC	Recommend
D4.172 Pelvis	8.21	GC	Recommend
D4.173 Calyces	8.05	GC	Recommend
D4.174 Adrenal glands	5.44	NC	Not recommend
D4.175 Spleen	8.55	VGC	Strongly recommend
D4.176 Right and left costophrenic angles	8.63	VGC	Strongly recommend
D4.177 Hepatorenal space (Morison’s pouch)	8.70	VGC	Strongly recommend
D4.178 Peri-splenic area for fluid	8.61	VGC	Strongly recommend
D4.179 Small bowel	7.13	SC	Recommend
D4.180 Subdiaphragmatic space	7.64	SC	Recommend
D4.181 Peristalsis	7.72	SC	Recommend
D4.182 Abdominal lymph nodes	5.69	NC	Not recommend
D4.183 Splenorenal	8.11	GC	Recommend
D4.184 Appendix	6.48	NC	Not recommend
D4.185 Ileocecal junction	5.61	NC	Not recommend
Pathology			
D4.186 Ascites	8.75	VGC	Strongly recommend
D4.187 Hemoperitoneum	8.47	VGC	Strongly recommend
D4.188 Hydronephrosis	8.56	VGC	Strongly recommend
D4.189 Sonographic Murphy Sign	8.18	GC	Recommend
D4.190 Cholelithiasis	8.49	VGC	Strongly recommend
D4.191 Gallbladder polyp	6.86	NC	Not recommend
D4.192 Splenic infarct	5.34	NC	Not recommend
D4.193 Hepatic hemangioma	4.63	NC	Not recommend
Pelvis			
Views			
D4.194 Urinary bladder, longitudinal	8.74	VGC	Strongly recommend
D4.195 Urinary bladder, transverse	8.67	VGC	Strongly recommend
D4.196 Uterus, transabdominal, long	8.63	VGC	Strongly recommend
D4.197 Uterus, transabdominal, trans	8.58	VGC	Strongly recommend
D4.198 Transvaginal scan	5.56	NC	Not recommend
Structures and physiology			
D4.199 Bladder, volume	8.18	GC	Recommend
D4.200 Uterus	8.53	VGC	Strongly recommend
D4.201 Fetal number	7.79	SC	Recommend
D4.202 Fetal heartbeat	8.16	GC	Recommend
D4.203 Fetal position	7.16	SC	Recommend
D4.204 Fetal size	6.74	NC	Not recommend
D4.205 Placenta	7.07	NC	Not recommend
D4.206 Testes	6.38	NC	Not recommend
D4.207 Epididymis	6.02	NC	Not recommend
Pathology			
D4.208 Free fluid	8.63	VGC	Strongly recommend
D4.209 Loss of ureteral jets	6.75	NC	Not recommend
D4.210 Hydroureter	6.86	NC	Not recommend
D4.211 Distended bladder	8.49	VGC	Strongly recommend
D4.212 Urolithiasis	6.85	NC	Not recommend
D4.213 Ovarian torsion	5.58	NC	Not recommend
D4.214 Testicular torsion	5.64	NC	Not recommend
D4.215 Foley catheter position	7.82	SC	Recommend
Head and neck			
Views			
D4.216 Longitudinal	8.51	VGC	Strongly recommend
D4.217 Transverse	8.45	VGC	Strongly recommend
Structures			
D4.218 Muscles of the neck	7.55	SC	Recommend
D4.219 Thyroid lobes	8.25	GC	Recommend
D4.220 Thyroid isthmus	7.95	SC	Recommend
D4.221 Parathyroid gland	5.27	NC	Not recommend
D4.222 Lymph nodes of the neck	6.77	NC	Not recommend
D4.223 Trachea	8.12	GC	Recommend
D4.224 Esophagus	7.71	SC	Recommend
D4.225 Globe of the eye	7.85	GC	Recommend
D4.226 Optic nerve	7.62	GC	Recommend
Pathology			
D4.227 Thyromegaly	7.20	SC	Recommend
D4.228 Thyroiditis	6.38	NC	Not recommend
D4.229 Thyroid mass or cysts	7.16	SC	Recommend
D4.230 Enlarged lymph nodes	6.81	NC	Not recommend
D4.231 Presence of endotracheal tube	7.13	SC	Recommend
D4.232 Esophageal intubation	7.27	SC	Recommend
D4.233 Eye globe	7.71	SC	Recommend
D4.234 Rupture of the globe	6.36	NC	Not recommend
D4.235 Papilledema	6.87	NC	Not recommend
D4.236 Transcranial Doppler	4.86	NC	Not recommend
D4.237 Retinal detachment	6.95	NC	Not recommend
D4.238 Foreign body of the eye	6.70	NC	Not recommend
Musculoskeletal			
Views			
D4.239 Views in general	8.43	VGC	Strongly recommend
D4.240 Transverse	8.46	VGC	Strongly recommend
D4.241 Longitudinal	8.46	VGC	Strongly recommend
Views, specific joints			
D4.242 Elbow, long	7.09	SC	Recommend
D4.243 Elbow, trans	7.00	SC	Recommend
D4.244 Wrist, long	7.09	SC	Recommend
D4.245 Wrist, trans	7.11	SC	Recommend
D4.246 Knee, long	7.74	GC	Recommend
D4.247 Knee, trans	7.65	GC	Recommend
D4.248 Ankle, long	6.84	NC	Not recommend
D4.249 Ankle, trans	6.79	NC	Not recommend
Structures, in general			
D4.250 Dermis and SC tissue	8.07	GC	Recommend
D4.251 Tendons	7.70	SC	Recommend
D4.252 Ligaments	7.48	SC	Recommend
D4.253 Cortex of bone	8.18	GC	Recommend
D4.254 Joint space	7.75	GC	Recommend
D4.255 Fat pads	7.46	GC	Recommend
D4.256 Synovium	7.14	GC	Recommend
Specific joint structures			
D4.257 Triceps tendon	6.87	NC	Not recommend
D4.258 Olecranon fossa fat pad	6.33	NC	Not recommend
D4.259 Distal radius	7.16	SC	Recommend
D4.260 Distal ulna	7.16	SC	Recommend
D4.261 Quadriceps tendon	7.29	SC	Recommend
D4.262 Bursa, suprapatella	7.27	SC	Recommend
D4.263 Patella	7.77	SC	Recommend
D4.264 Patellar tendon	7.70	SC	Recommend
D4.265 Tibial tuberosity	7.15	SC	Recommend
D4.266 Achilles tendon	7.58	SC	Recommend
D4.267 Distal fibula	7.11	SC	Recommend
D4.268 Distal tibia	7.04	SC	Recommend
D4.269 Talo-Fib ligaments	5.94	NC	Not recommend
D4.270 Talo-Tib ligaments	5.91	NC	Not recommend
D4.271 Shoulder humeral head	7.59	GC	Recommend
D4.272 Shoulder glenoid	7.07	SC	Recommend
D4.273 Shoulder acromion	6.85	NC	Not recommend
D4.274 Shoulder clavicle	7.28	SC	Recommend
D4.275 Shoulder biceps tendon	7.17	SC	Recommend
D4.276 Shoulder supraspinatus tendon	6.87	NC	Not recommend
Pathology			
D4.277 Joint effusions	8.28	GC	Recommend
D4.278 Bursal fluid	7.57	GC	Recommend
D4.279 Calcium deposition	6.49	NC	Not recommend
D4.280 Soft tissue edema/cobblestoning	7.96	GC	Recommend
D4.281 Soft tissue abscess or cyst	8.19	GC	Recommend
D4.282 Soft tissue solid mass	7.47	SC	Recommend
D4.283 Clubbing of the fingers	5.48	NC	Not recommend
D4.284 Carpal tunnel—median nerve	6.51	NC	Not recommend
D4.285 Joint dislocation	6.30	NC	Not recommend
D4.286 Tendon impingement syndrome	5.67	NC	Not recommend
D4.287 Tendonitis	6.20	NC	Not recommend
D4.288 Complete tendon tear	6.77	NC	Not recommend
Part III. Procedures/protocols			
Procedures			
D4.289 Peripheral vein cannulation (PVC)	8.23	GC	Recommend
D4.290 Central venous cannulation (CVC)	7.59	SC	Recommend
D4.291 Pericardiocentesis	6.44	NC	Not recommend
D4.292 Paracentesis	7.29	GC	Recommend
D4.293 Thoracentesis	7.23	GC	Recommend
D4.294 Arthrocentesis	7.04	SC	Recommend
D4.295 Lumbar puncture	6.71	NC	Not recommend
D4.296 Visualize any body cavity/fluid collection before needle	7.95	GC	Recommend
D4.297 We should not add specific skills	5.87	NC	Not recommend
D4.298 Students should be able to use ultrasound to visualize fluid-filled cavities	8.75	VGC	Strongly recommend
D4.299 Students should be able to use ultrasound to guide a needle safely into a fluid-filled cavity, as demonstrated on patients or a phantom model	8.59	VGC	Strongly recommend
Protocols			
D4.300 E-FAST protocol	7.75	GC	Recommend
D4.301 RUSH protocol	6.88	SC	Recommend
D4.302 CLUE protocol	6.14	NC	Not recommend
D4.303 BLUE protocol	6.39	NC	Not recommend
D4.304 Students do not need to learn specific ultrasound protocols	4.80	NC	Not recommend

### Statements and discussion

There were a total of 332 consensus conference statements and curricular content items in Domains 1–4. Of these, 145 were recommended, 126 were strongly recommended, and 61 were not recommended. Relevant conference discussion, written survey comments of participants, and more recent references have been included in the discussion of the final consensus recommendations.

### Domains 1–3

Of the 28 statements in Domains 1–3 covering the scope, the rationale, and the characteristics of an undergraduate ultrasound curriculum, 19 statements were recommended and 9 were strongly recommended. As anticipated, GRADE evaluation of the literature did not demonstrate a high level of evidence for the statements, confirming the need for an emphasis on expert opinion.

These 28 consensus statements can serve as a guide for medical school curriculum directors and their institutions in the planning, development, and expansion of ultrasound medical student education. Details including statements, rationales and relevant references of all 28 statements can be found in Additional file 1: Appendix S1. The nine statements that the expert panelists strongly recommended are highlighted here as well as one of the recommended statements of particular significance related to non-physician ultrasound education.

Domain 1: scope of consensus conference curriculum

**D1.1**: The ICC will produce consensus recommendations on “An integrated ultrasound curriculum” (“curriculum”) for undergraduate medical education (medical school).

The overall structure of the medical student curriculum should be that of an integrated curriculum across concurrent courses horizontally and across courses and clinical clerkships vertically for each year of medical school. Integration can be broadly defined operationally as deliberately unifying separate areas of knowledge [26]. Globally, medical education accrediting bodies have encouraged and even required that medical school curricula be integrated [27–29]. The Carnegie Foundation Report in 2010 *Educating Physicians: A Call for Reform of Medical School and Residency* calls for more integration throughout medical education [31]. Various levels of integrated ultrasound curricula have been successfully implemented in medical schools internationally varying in size, school mission, and integration format [33–38, 157, 173].

**D1.2**: The curriculum forms the foundation for ultrasound as a core clinical competency for all graduates regardless of specialty choice.

Over the past two decades, competency-based medical education (CBME) has become the standard for medical education. Competency can be defined as an observable, measurable, and assessable ability of a health professional. Competencies can be broken down into milestones that are observable steps used to assess and document a learner’s progress toward a given competency along a developmental continuum [39, 40].

General Physician Competencies have been clustered into domains of competence which are broad but distinguishable areas of competence that constitute a general descriptive framework for a profession [41]. From the work on competencies and domains have come Entrustable Professional Activities (EPAs). EPAs are units of professional practice, defined as tasks or responsibilities that trainees are entrusted to perform unsupervised once they have attained sufficient specific competence [42, 43].

Ultrasound is well suited for a competency-based model of medical education and EPAs. Ultrasound can directly serve as a competency component for a number of the core EPAs such as performing a quality physical examination (EPA 1), prioritizing a differential diagnosis following a clinical encounter (EPA 2), recommending and interpreting common diagnostic and screening tests (EPA 3), recognizing a patient requiring urgent or emergent care and initiating evaluation and management (EPA 10), and performing general procedures of a physician (EPA 12) [3, 44–52].

In addition to these direct roles that ultrasound can play in these EPAs, it can also play important indirect roles in several other core EPAs such as being more knowledgeable about ordering imaging studies (EPA 4), forming clinical questions (EPA 7), collaborating on an inter-professional team (EPA 9), understanding informed consent (EPA 11), and contributing to a culture of safety and improvement (EPA 13).

Patient safety is an important aspect of EPAs as it has been proclaimed as “the primary motivation for the work on EPAs” [42]. Because ultrasound does not use ionizing radiation like X-rays and computed tomography, it is a particularly safe imaging modality. In addition, the Agency for Healthcare Research and Quality (AHRQ) has identified the use of real-time ultrasound guidance during central line insertion as a top ten patient safety practice. The AHRQ also recommends that providers not delay in adopting this practice of using ultrasound guidance [53].

Domain 2: rationale for the curriculum

**D2.8:** The curriculum enhances the overall educational experience.

Early POCUS research on medical student exposure to ultrasound focused primarily on student satisfaction and found that students enjoy having ultrasound in the curriculum and feel it enhances their education [33, 70, 82, 84, 116–119]. However, some evidence suggests that students can feel overconfident in their POCUS skills or image interpretation at a time when they have limited understanding of the underlying core principles of patient management leading to the consideration that POCUS might best be considered as a supplemental skill [120]. POCUS has been described as motivating students to delve deeper into matters of interest while not appearing to adversely impact the time necessary to learn the content that already exist in overcrowded undergraduate curricula [35, 121]. Although there is some suggestion that ultrasound improves basic science knowledge and clinical skill, future educational research will need to focus more on objective outcomes that show that ultrasound enhances learning of content and prepares students for advanced training and clinical practice.

**D2.9**: Medical students can learn basic ultrasound.

There is ample evidence that students can learn basic ultrasound and ultrasound applications, including both image acquisition and image interpretation [35, 70–72, 75, 122–142]. Image integration into clinical practice still requires clinical knowledge that exposure to ultrasound anatomy and physiology alone does not confer. Once a standardized ultrasound curriculum is established, more individual and collaborative research efforts will be needed to further define the best methods of ultrasound instruction and assessment of student ultrasound knowledge and skill.

Domain 3: characteristics of the curriculum

**D3.1**: The ultrasound curriculum forms the foundation for ultrasound training along a continuum of medical education from undergraduate through graduate to continuing medical education.

Point-of-care ultrasound at the patient's bedside represents a new tool for the practicing physician. Originally introduced by those caring for emergency and critical care patients to rapidly evaluate and manage their patients, its use has spread throughout hospital services and outpatient care settings. As many as 20 US medical and surgical specialties now require competency and/or experience in ultrasound applications at the completion of their graduate medical education training [152].

Because POCUS is rapidly diffusing into medical practice, it is essential that there be a structured and well-organized program to facilitate ultrasound training in schools of medicine and a smooth transition to postgraduate training.

A recent scoping review of the literature on ultrasound in medical school education and a consensus of ultrasound education directors support the need for a standardized point-of-care ultrasound curriculum that would lead to the development of common standards for milestones and competency-based assessments [19, 155]. Hence, a standardized foundational curriculum delineated by experts in the field of ultrasonography, by those experienced in its use in diverse clinical settings and at the point of patient care, and by educators knowledgeable about the trajectory of physician development can provide guidance as this new skill is integrated into the profession throughout the world.

**D3.8**: The ultrasound curriculum enhances the learning of clinical sciences through the integration of ultrasound into clinical problem solving.

Along with the integration of the patient history, the physical exam, and laboratory data, point-of-care ultrasound can provide additional information readily available at the time of the patient encounter leading to a more rapid and accurate guide to diagnosis and treatment [3, 163]. Thus, the introduction of ultrasound into the medical school curriculum, likewise, may provide additional accuracy in the accumulation of patient information that fosters improved understanding of underlying pathophysiology. Such improved understanding can aid in the development of a student’s rational diagnostic or therapeutic plan. Ultrasound in undergraduate medical education has been shown to improve the accuracy of the student physical examination. For example, students with limited ultrasound training were more accurate than cardiologists in cardiac exams [44]; than faculty in estimating the size of the liver [45]; and in locating the femoral artery with than without ultrasound [99]. Integration of ultrasound has the potential to improve other aspects of the physical exam, including evidence of professionalism [154]. Use of ultrasound by students may enhance their ability to assess patients with critical presentations, such as hypotension [100]. Accurate patient assessment during physical examination allows the student to better integrate findings into their overall clinical problem solving.

The following recommendations are clustered for discussion as all three relate to the value and validity of the recommended curriculum in the context of organized medical ultrasound.

**D3.12**: The ultrasound curriculum is based on evidence and expert opinion.

**D3.13**: The ultrasound curriculum is consistent with recommendations and guidelines of well-established specialty organizations.

**D3.14**: The ultrasound curriculum is consistent with recommendations and guidelines of regulatory bodies with significant experience in ultrasound.

Point-of-care ultrasound represents a new clinical skill with much information now accumulating on its applicability to many areas of medicine. As such, a burgeoning literature along with expert opinion is becoming widely accessible to guide the development of an international curriculum. A number of professional societies have developed or are developing guidelines and/or curricula in the area of ultrasound [5, 106, 164–172]. The International Consensus Curriculum aligns with these societal guidelines to prepare early learners with the necessary foundation to use POCUS in their future chosen area of medicine, as supported by the guidelines of these national and international societies.

In addition to these strong recommendations from Domains 1–3, recommended statement D1.4 concerning the role of the consensus conference curriculum in non-physician education warrants some clarification based on considerable conference meeting discussion and survey comments.

**D1.4:** The curriculum can serve as a valuable resource for the development of ultrasound training programs for non-physician healthcare providers such as advanced nurse practitioners and physician assistants.

Considering the overlap in medical student educational content and skill with that of other healthcare professionals as set by their accrediting bodies such as nurse practitioners, nurses, physician assistants, and emergency medicine technicians, an integrated ultrasound curriculum for medical students should prove to be a valuable and appropriate resource for the education of these and other healthcare professionals [27, 59–62]. It has been demonstrated that non-physician providers can learn and competently use ultrasound in the clinical setting [63–66]. In addition, a common clinical skill like ultrasound offers excellent opportunities for inter-professional training.

There was agreement in conference discussions that a standardized ultrasound curriculum for medical students determined by this consensus conference could be a valuable resource for non-physician healthcare providers. However, it was emphasized that the curriculum should not be considered a recommended curriculum; it should only serve as a resource for curricular development. Other healthcare providers will need to determine the specifics of their ultrasound curricula based on their accreditation and clinical practice standards as determined by their own professional organizations.

Domain 4: curricular content

Domain 4 focused on the content of a medical student ultrasound curriculum. Of the 304 Domain 4 content items, 126 (41.4%) were recommended, 117 (38.5%) were strongly recommended, and 61 (20.1%) were not recommended. All recommended content would be considered appropriate for a medical student ultrasound curriculum, but should not be considered as required content. Content used within an individual medical student curriculum should be based on a number of factors including how well the specific content items fulfill the needs and objectives of the courses and clinical clerkships in the curriculum, the availability of adequate resources to implement the specific ultrasound components, and the faculty expertise available to teach the specific components of the ultrasound curriculum.

It should also be noted that for those medical educational systems that have medical school graduates immediately engaged in various levels of independent clinical practice, assessment of medical student ultrasound competency at graduation would need particular attention. Completion of the recommended ultrasound curricular content does not ensure independent clinical ultrasound competency. The decision of practice competency directly after medical school graduation will need to be made by the individual medical school and/or the appropriate accrediting body in accordance with established clinical practice standards.

Medical schools with successful ultrasound programs have generally started by introducing a small number of basic ultrasound components into the curriculum and have then expanded the number of ultrasound components over time [33, 37, 38, 157]. It is important to not overwhelm faculty and students with new material on ultrasound to assimilate into an already crowded curriculum. An incremental approach also allows time to gather student and faculty feedback evaluating the program as it develops so that informed curricular management decisions can be made.

*Domain 4 part 1:* basic foundations of point-of-care ultrasound

Part one of Domain 4 contained 92 content items related to the “Basic Foundations of Point-of-Care Ultrasound”. These items covered the physics of ultrasound, imaging modalities, ultrasound terminology, machine and probe characteristics, image acquisition, basic image interpretation, patient care issues, and correlation of clinical findings.

Of the 92 items, 26 (28.3%) were recommended, 64 (69.6%) were strongly recommended, and 2 (2.2%) were not recommended. The two items not recommended were related to the acquisition of images with power Doppler (D4.74) and spectral Doppler (D4.75) imaging modalities. Although it was recommended that students should understand the fundamental principles of power Doppler and spectral Doppler, it was felt that image acquisition with these two modalities was too advanced for medical student ultrasound education.

The 90 basic foundation items recommended or strongly recommended are consistent with the essentials and standards for education in medical sonography across multiple ultrasound societies and accrediting bodies [174–179]. These recommended basic items should help promote the standardization of medical student ultrasound education globally as well as provide a common language and framework to enhance communication among those interested in ultrasound education, practice, and research. This will be particularly helpful as collaborative efforts develop across the continuum of ultrasound education from undergraduate to postgraduate medical education. Further strengthening the continuum of ultrasound education with standardization of the basics will allow directors of postgraduate medical education to anticipate the ultrasound knowledge and skill levels of incoming medical school graduates and plan a smooth transition to postgraduate training.

Several topics and items within Domain 4 Part 1 deserve special comment. The first of these concerns “proper care for the patient” which focuses on patient interactions that include professional communication (D4.85), informed consent (D4.86), privacy (D4.87), comfort (D4.88), patient positioning D4.89), and documentation (D4.90). These strongly recommended patient interactions should be at the core of medical student education and taught, modeled, and assessed from the earliest stages of teaching ultrasound to students. With ultrasound education, the patient’s well-being should always be the primary focus of the patient encounter and not become secondary to the technology. One of the significant advantages of ultrasound education is a greater return to the patient’s bedside offering many opportunities to teach and model the art, the science, and the humanity of practicing medicine. Spending more time with the patient at the bedside is consistent with initiatives to foster more meaning and joy in work and deeper engagement with patients [180].

In addition to these recommendations, two other patient-centered recommendations related to patient safety need special emphasis. Specifically related to patient safety were strong recommendations for the principle of using ultrasound intensity as low as reasonably achievable, known as the ALARA principle, (D4.91) and the importance of appropriate cleaning and disinfection of probes (D4.27) prior to scanning. This recommendation of probe disinfection has taken on an even greater significance during the COVID pandemic with acute concern for transmission of infection during ultrasound procedures. Portable bedside ultrasound has played a significant role in the diagnosis and management of COVID patients across the globe [181]. In addition, the ability to more easily clean and disinfect these portable bedside devices rather than the larger cart-based machines and those in the radiology suite and limiting the need to transport patients throughout the medical facility for imaging will likely improve protection against transmission of infection to non-COVID patients, staff, and healthcare providers.

Also worthy of special note in Domain 4 Part 1 is the importance of correlating ultrasound images with clinical findings (D4.92). This statement received the highest mean appropriateness score (8.95) of all statements in the survey and reflects the high priority the voting panelists place on the educational value of ultrasound as an important tool to better understand medicine and improve clinical care.

An issue under “Basic Foundations” of ultrasound that generated significant discussion and comments was related to transducer or probe manipulation terminology (D4.28-D4.33). Even though all six manipulation items were recommended or strongly recommended, a number of panelists commented on a preference for specific transducer manipulation terms while scanning and expressed the need for more standardization of probe manipulation terms to enhance consistency of hands-on scanning instruction.

Probe manipulation terminology has been a controversial issue for years as multiple terms have been used for the same or similar manipulations of the probe such as “fan” or “tilt” the probe. These terms have been variably adopted by ultrasound users and educators and can be a source of confusion to new learners who are being taught by various instructors using different terms for the same probe maneuver. It can also be a source of confusion when students are viewing instructional videos that use different terminology from what they have learned. Comments from the panelists were mixed on this topic with some experts recommending that an effort be made for a universally accepted set of terms while others felt a group of acceptable terms could be recommended and individuals in various educational programs could decide which ones they wish to use coincident with local use. For the consensus conference, it was decided to use six probe motions that have been well-described in the literature [182]. Even though it is unlikely that a single set of probe manipulation terms will be universally adopted from this consensus process, these recommendations may encourage movement toward a more uniform set of terms.

*Domain 4 part 2:* views, structure/physiology, pathology

Domain 4, Part 2 items relate to specific ultrasound views, structures/physiology, and pathology with regional and organ subdivisions of heart and vessels, lungs and chest, abdomen, pelvis, head and neck, and musculoskeletal. Of the 196 items, 92 (46.9%) were recommended, 51 (26.0%) were strongly recommended, and 53 (27.1%) items were not recommended.

### Ultrasound views

There was very good agreement on teaching students ultrasound views proposed by the Domain 4 task team and the expert voting panel. Of the 30 views, 10 (33.3%) were recommended, 17 (56.2%) were strongly recommended, and 3 (10.0%) were not recommended.

The recommended and strongly recommended views include widely recognized standard views of the various organ systems. The transvaginal view of the pelvis (D4.198) was not recommended as it was felt to be more appropriate for postgraduate medical education. In addition, cultural differences were also noted with respect to training students in the transvaginal view and it was felt that if the transvaginal view is taught, it should be done on simulators and not patients. The other two views not recommended were two specific ankle views (D4.248–249) that were not felt to be of significant value to warrant having students learn them in medical school.

Recommendations on what ultrasound views to teach students are critically important, especially early in the ultrasound learning process. Introductory views should be relatively easy to learn for those new to ultrasound. They should also allow students to visualize anatomical structures and physiological organ functioning important in understanding normal anatomy, normal physiology, and common pathophysiology to prepare them well for postgraduate training.

A limited number of more advanced views can be taught in medical school, but it would not be practical to teach students all ultrasound views in medical school due to the time required. Should a school wish to offer more advanced ultrasound scanning skills for students, several elective options can be considered. These include an independent ultrasound study month, departmental ultrasound offerings, participation in ultrasound research, and final year compressed or boot camp ultrasound experience to prepare students for specific residency ultrasound applications [21, 33, 68, 183–185]. Another option that allows interested students to gain more advanced ultrasound skills is through student ultrasound interest group activities which generally occur outside of the formal curricular schedule [186].

It should be noted that even with standard basic ultrasound views, some of these views are easier to learn than others, such as the parasternal long axis (PLAX) view of the heart as compared to the apical 4 chamber view of the heart. Once the PLAX view is learned and practiced, learning the apical 4 chamber view is generally much easier. Thus, it is best to start with relatively easy to learn views and progress to slightly more difficult views over time. This same approach is also true in considering the scanning difficulty level of models and patients used for ultrasound instruction. Starting with relatively easy-to-scan models and progressing to more difficult-to-scan models creates a better learning experience. This approach allows students to progressively improve their basic scanning skills and confidence. It also allows them to more efficiently capture quality images of the important structures and organs under study to enhance learning of the primary course content material.

### Structures/physiology

There was good agreement between Doman 4 task team proposed structure and physiology content and the expert voting panel. Of the 94 structure/physiology items, 52 (55.3%) were recommended, 21 (22.3%) were strongly recommended, and 21 (22.3%) were not recommended.

Similar to the considerations for what ultrasound views to teach, the specific structures and physiology to teach with ultrasound should be based on their value in learning normal anatomy and physiology and preparing students to better understand pathophysiology important to the practice of medicine. They also need to be appropriate for the undergraduate level of medical education. More advanced content should be left for postgraduate medical education or offered in student electives for those wanting to learn more than what is offered in the required student curriculum.

The voting panel did not recommend the 21 content items for three primary reasons. From international conference discussion and panelists’ written comments, some of the required ultrasound images were considered too difficult for students to consistently visualize well enough for them to be used to teach the course content such as the adrenal glands (D4.174), the pancreas (D4.168), and the appendix (D4.184). Some structures and physiology were just not considered appropriate for a medical student basic curriculum such as the placenta (D4.205) and testes (D4.206). Finally, it was felt that topics with multiple appropriate examples in the same class of structures, such as peripheral blood vessels and musculoskeletal structures, should not be covered comprehensively, but instead one or two examples should be taught. For example, students could learn common musculoskeletal joint structures and biomechanical principles by learning to scan the knee without spending additional time scanning multiple other joints.

### Pathology

The final section of Part 2 was concerned with what pathology to teach medical students with ultrasound. This section had a relatively low level of agreement between what the Domain 4 task team proposed for curricular content and what the expert panel felt was appropriate for medical student education. Of 70 pathology items only 30 (42.9%) were recommended, 11 (15.7%) were strongly recommended, and 29 (41.4%) were not recommended.

This low level of recommendation was not related to the value of ultrasound in teaching pathology, but rather to the specific ultrasound pathology content. Much of the pathology was felt to be more appropriate for postgraduate training as opposed to medical student education. There was also some concern expressed by panelists that students could become overconfident in their ability to identify or rule out pathology with ultrasound. This could have serious adverse consequences for patients such as pursuing additional unnecessary and costly testing with potential risk or not recognizing significant clinical findings, thus delaying diagnosis and treatment. An example of this type of pathology that was not recommended to be included was dissection of an abdominal aortic aneurysm (D4.135).

While not included as content for voting in this international consensus conference, the notion of overconfidence, knowing one's limitations in ultrasound and medical knowledge, as well as, understanding the inherent limitations of ultrasound in specific circumstances should be addressed in the curriculum as a whole. These aspects of ultrasound education could be clustered as learning the indications, limitations, benefits, and risks of ultrasound in common clinical scenarios [19].

Less consensus for recommending the pathologies to teach medical students may have been partly related to the diverse composition of the voting panelists. Different specialists and subspecialists would likely differ in the value they place on various pathologies to teach medical students and the ability of students to adequately capture and interpret those ultrasound images during medical student education.

Under pathology, it is instructive to note that the Sonographic Murphy Sign (D4.189) is a good example of ultrasound enhancing the physical examination (D2.5). Sonographic Murphy sign is a painful reaction of the patient when pressing directly over the gallbladder with the ultrasound probe which could be consistent with acute cholecystitis. Being able to look under the skin with ultrasound to provide visual information can enhance the accuracy of the physical examination as well as enhance learning of physical examination skills by providing real-time validation of the physical examination component. Ultrasound can be applied to learning many aspects of the physical examination such as confirming inspection of the neck for the location and size of the thyroid, palpating the liver and gallbladder for location, size, and tenderness, percussing the lungs for the resonance of normal lung or the dullness of a pleural effusion, and auscultating the heart for a murmur or a pericardial friction rub. [33, 135, 154].

*Domain 4 part 3:* procedures and protocols

Domain 4, Part 3 items relate to ultrasound procedures and protocols and it also had a relatively low level of agreement between the Domain 4 task team proposals and the expert voting panel. Of 16 items, 8 (50.0%) were recommended, 2 (12.5) were strongly recommended, and 6 (37.5%) were not recommended.

The skill of ultrasound-guided procedures was robustly discussed during face-to-face consensus conference meetings and a number of written comments appeared on the voting survey. It was strongly felt that students should be taught to visualize fluid-filled cavities with ultrasound (D4.298) and how to use ultrasound to guide a needle safely into a fluid-filled cavity (D4.299). A number of common guided procedures were recommended, including peripheral (D4.289) and central line cannulation (D4.290), paracentesis (D4.292), thoracentesis (D4.293), and arthrocentesis (D4.294). However, the less common and more risky guided procedures of pericardiocentesis (D4.291) and lumbar puncture (D4.295) were not recommended. However, it was also expressed that how to use ultrasound to guide a needle or catheter was the important skill and there was no need to learn multiple guided procedures. Learning a variety of guided procedures was best reserved for postgraduate medical training when the focus could be on procedures more relevant to the specialty pursued. It was also expressed that, in general, learning guided procedures is best done on phantom models and not on live subjects.

The final category of Part 3 was ultrasound protocols and included the more common clinical protocols. Two of these were recommended for medical student curricula. These included the E-FAST protocol (Extended Focused Assessment with Sonography in Trauma) (D4.300) for trauma and the RUSH protocol (Rapid Ultrasound for Shock and Hypotension) (D4. 301) for hypotension and shock. Other protocols were not recommended. There was general discussion and panelists commented that individual components of protocols could be taught, but there would not be significant value in teaching more than one or two protocols given the many and continually expanding list of protocols. Most protocols are best left for advanced training where specific protocols related to various specialties could be learned and clinically applied.

It should be noted that both the E-FAST and RUSH protocols have been used for teaching medical student content such as physiology clinical correlation and trauma assessment in emergency medicine and surgery [33, 34, 187]. Thus, these two protocols could serve as valuable teaching protocol examples should a school wish to introduce a few select protocols consistent with their curricular objectives.

### Consultant and student/resident survey responses

There was overall good agreement with the survey results of the voting panelists and the consultants using mean appropriateness scores. Some minor differences of note were higher scores from the consultants than the voting panelists for the role of ultrasound in self-directed learning (D3.10)—8.44 versus 7.83 and life-long learning (D3.11)—8.42 versus 7.62. The highest score for both groups was for the importance of correlating ultrasound images with clinical findings (D4.92)—8.98 consultants, 8.95 voting panelists.

A high score for correlation with clinical findings was also recorded by the students/residents (8.88), but their highest score (8.94) went to multiple content items on the basics of scanning and concern for the patient. In general, students/residents gave higher scores for pathology items in the curricula content than the voting panelists. For example, appropriateness scores for abdominal aortic aneurysm dissection (D4.135) for panelists was 7.07 and 8.06 for the students/residents. The students also gave higher appropriateness scores for ultrasound procedures and ultrasound protocols. Because of the small, self-selected nature of the student/resident survey participants, definitive conclusions cannot be drawn about student/resident opinions, but it does suggest a difference of opinion in some areas of ultrasound education. These potential differences should be explored and students and residents should be included in curricular development.

Additional comments from consultants for the most part echoed those of the voting panelists including the need for balance of ultrasound content in an already crowded medical student curriculum and remaining within the appropriate level of knowledge, attitude, and skills for medical students, especially with respect to advanced scanning techniques, pathology, and protocols. Students did mention that the curricular content appeared to be comprehensive and would require strong medical school buy-in to be successful.

## Consensus conference conclusions

A sense of urgency exists for the need to incorporate ultrasound into medical student education. The data for the value of ultrasound to improve the quality of patient care, patient safety, and access to care for all patients across the globe have been mounting for almost three decades. The technological advances and lower cost have made ultrasound highly accessible and the interest in ultrasound education in medical school is rising at an exponential rate. The adoption of ultrasound across postgraduate (residency) programs is rapidly increasing and the calls for help in developing the appropriate educational support are growing louder as echoed in a recent POCUS article in the *New England Journal of Medicine* [23].

It is imperative that the undergraduate medical education community proceed in a timely fashion with a plan to help ensure ultrasound training for medical students that is appropriate, supported with ongoing quality research efforts, and offers a smooth transition to postgraduate training. Establishing a standardized ultrasound curriculum based on the available evidence and global expert opinion is a critical step in that process. This conference was designed to address this need. All recommended and strongly recommended statements of curricular content are listed in Additional file 2: Appendix S2.

The international conference addressed several limitations of previously reported consensus recommendations for foundational ultrasound curricula for medical student education [155, 158, 188]. These earlier recommendations often were directed at specific clinical components rather than an integrated curriculum that spans basic and clinical sciences of undergraduate medical education. By necessity, these recommendations usually engaged a national or regional approach, as opposed to the global approach taken here. Previous consensus conferences often lacked expertise across clinical specialties, subspecialties, and basic science disciplines, a much-needed perspective for an integrated curriculum. Likewise, there has also been limited representation across global educational systems which not only vary in location but also culture, curricular models, available resources, educational accrediting standards, and institutional vision and mission [189]. In addition, some of the earlier publications used a less comprehensive consensus methodology than was used here, which includes quantifying the level of evidence of the relevant literature and making that available to the voting participants.

To overcome these various limitations, especially those related to the diversity of expert ultrasound practitioners and educators, a large, diverse group of 64 expert voting panelists representing over 20 specialists, subspecialists, and basic science educators from 16 countries were selected to participate. Over 90% of panelists voted in both rounds, ensuring recommendations from a diversity of panelists. Also, contributing expert input to the consensus process were 50 global consultants with similar ultrasound credentials as the voting panelists as well as 21 medical students and residents with a keen interest in ultrasound education. An extensive multi-source literature search and a rigorous modified Delphi methodology were utilized including the GRADE method to evaluate level of evidence and the RAND methodology for degree of appropriateness, consensus, and final recommendations.

In addition to the formal voting results for each statement, relevant concerns, comments, and advice from the consensus participants have been included in the discussion. These comments provide valuable insight from those who have extensive experience in ultrasound education and can further assist those new to medical student ultrasound education in implementing an ultrasound curriculum. Also included in the discussion are the results of more recent publications on ultrasound education. All voting panelists reviewed the journal manuscript for accuracy of content prior to submission. This consensus conference represents the most comprehensive medical school consensus process to date to standardize a global ultrasound curriculum.

There were several limitations of the consensus process that should be noted. Despite the broad representation of clinical specialties and subspecialties, not all areas of medicine were included (e.g., ophthalmology and physical medicine) that might have considerations for future ultrasound practice. Additional representation of basic biomedical science, pathology, and even genetics could provide an even broader perspective. Likewise, a broader global ultrasound education perspective should be considered in the future (i.e., Africa). While students and residents were included, more systematic inclusion of these stakeholders will likely be more feasible as ultrasound education spreads throughout institutions. This input will prove more helpful when teaching and assessment methods are critically addressed in future research and consensus processes.

It is hoped that the consensus curriculum will facilitate independent and collaborative research into what aspects of the proposed curriculum work well and what should be modified, added, or eliminated. The curriculum should be considered an ongoing global educational project. It will need to be updated as new ultrasound technology, ultrasound applications, and research-based educational and clinical results and advances become available. A standardized curriculum should enhance collaboration among directors of undergraduate and postgraduate medical education to strengthen the continuum of ultrasound education and help ensure the smooth transition from one stage of training to the next and advance patient care.

Currently medical student ultrasound education, including hands-on scanning instruction, is supported by the ultrasound specialties of radiology, cardiology, obstetrics and gynecology and major national and international ultrasound organizations with publications, meetings, student interest groups, and online education material on their websites [158, 175–179, 190–193]. Collaborative efforts with these established ultrasound groups will help modify and advance this curriculum, as well as strengthen the continuum of ultrasound education across the professional lives of healthcare providers.

Ultrasound presents an opportunity in our lifetimes to improve how we fundamentally teach and practice medicine to the benefit of students and patients across the globe. In the rich humanitarian tradition of medicine, may we seize this opportunity for teaching and using ultrasound to make this world a healthier and better place for all.

## Supplementary Information

**Additional file 1: Appendix S1. **International Consensus Conference Statements, Rationale, and References for Domains 1-3.

**Additional file 2: Appendix S2. **Recommended medical student ultrasound curricular content.

## Data Availability

The data sets used and analyzed during the current study are available from the corresponding author on reasonable request.
