# Supplementary material for: International consensus conference recommendations on ultrasound education for undergraduate medical students
Source: Ultrasound J. 2022 Jul 27;14:31. doi: 10.1186/s13089-022-00279-1 (PMC9329507; doi:10.1186/s13089-022-00279-1)
Supplement: Supplementary file 1 — Additional file 1: Appendix S1. International Consensus Conference Statements, Rationale, and References for Domains 1-3. [file 13089_2022_279_MOESM1_ESM.docx]

**Appendix 1**

**International Consensus Conference Statements, Rationale, and References for Domains 1-3**

**Domain 1. Scope of Consensus Conference Curriculum**

- 1. **The ICC will produce consensus recommendations on “An integrated ultrasound curriculum” (“curriculum”) for undergraduate medical education (medical school).**

Integration can be broadly defined operationally as deliberately unifying separate areas of knowledge (1). Interest in an “integrated curriculum” in medicine has grown tremendously in the past two decades and a number of global medical education accrediting bodies have encouraged and even required that medical school curricula be integrated (2-4). In addition, one of the primary “calls” in the Carnegie Foundation Report in 2010 Educating Physicians: A Call for Reform of Medical School and Residency is for more integration throughout medical education (5).

An integrated curriculum is consistent with the principles of adult learning theory and “meaningful learning” that will later be applied, organizing knowledge to match the way it is to be used, and the transfer of learning with comparison of basic science concepts and clinical examples (6).

Due to the many challenges of designing studies to test the effectiveness of an integrated curriculum, there is not much published on the effectiveness of integrated curricula but outcome trials that do exist have shown “at least non- inferior, if not objective benefits, for learners in an integrated setting.” (5)

Von der Veken, et al have demonstrated the value of an integrated curriculum (ICMC) as compared to a conventional discipline-based curriculum (CMC) for general medical education over a 6 year period with 1421 medical students using an independent annual knowledge acquisition exam (Dutch Inter-University Program Test) for basic and clinical sciences (7). Students in the ICMC out- performed CMC students at all curriculum years 2-6 for both basic and clinical sciences (P<0.01) and for all clinical sciences years (P<0.001).

Integrated ultrasound curricula have been successfully implemented in a number of medical schools varying in size, school mission, and integration format (8,10,13).

Most programs report a high degree of student satisfaction with the ultrasound component of the education program and those that report knowledge and/or skills acquisition report a moderate to high level of achievement.

Hoppmann, et al report over an 8-year period of an integrated ultrasound curriculum that first year and second year medical students averaged 96% correct assessment on hands-on ultrasound objective structured clinical exams (OSCEs) (8).

Rao, et al report at the end of a one year ultrasound pilot program for first year medical students that volunteers who completed the ultrasound experience (N=110), scored 87% correct on a hands-on phantom ultrasound exam (10).

Wilson, et al report at the end of a 4-year POCUS curriculum that fourth year medical students scored 77% correct on a 61 question multiple-choice examination that included ultrasound video loops testing knowledge and image interpretation (13).

**References**

1. Goldman E, Schroth WS. Deconstructing integration: A framework for the rational application of integration as a guiding curricular strategy. Acad Med 2012;87(6):729-734.
2. Liaison Committee on Medical Education (LCME). 2017. Functions and structure of a medical school: Standards for accreditation of medical education programs leading to the MD Degree. Washington, DC. USA. <http://lcme.org/publications/>
3. Australian Medical Council Limited. Standards for assessment and accreditation of primary medical programs by the Australian Medical Council 2012. Kingston, ACT. Australia. <http://www.amc.org.au/files/d0ffcecda9608cf49c66c93a79a4ad549638bea0_original.pdf>
4. The Association of Faculties of Medicine of Canada (AFMC). The future of medical education in Canada (FMEC): A collective vision for MD education. 2009. Ottawa, ON. Canada. <https://www.afmc.ca/future-of-medical-education-in-canada/medical-doctor-project/pdf/FMEC_CollectiveVisionMDEducation_EN.compressed.pdf>
5. Brauer DG, Ferguson KJ. The integrated curriculum in medical education: AMEE Guide No. 96. Med Teach 2015;37:312-322.
6. Irby DM, Cooke M, O’Brien BC. Calls for reform of medical education by the Carnegie Foundation for the Advancement of Teaching; 1910 and 2010. Acad Med 2010;85(2):220-227.
7. Van der Veken J, Valcke M, De Maeseneer J, et al. Impact of knowledge acquisition of the transition from a conventional to an integrated contextual medical curriculum. Med Educ 2009;43:704-713.
8. Hoppmann R, Rao V, Bell F, et al. The evolution of an integrated ultrasound curriculum (iUSC) for medical students: 9-year experience. Crit Ultrasound J. 2015;7(1):18-33.
9. Palma JK. Successful strategies for integrating bedside ultrasound into undergraduate medical education. Military Medicine 2015;4:153-157.
10. Rao S, van Holsbeeck L, Musial JL, Parker A, Bouffard JA, Bridge P, Jackson M, Dulchavsky SA (2008) A pilot study of comprehensive ultrasound education at the Wayne State University School of Medicine: a pioneer year review. J Ultrasound Med 27(5):745-749.
11. Russ BA, Evans D, Morrad D, et al. Integrating point-of-care ultrasonography into the osteopathic medical school curriculum. J AM Osteop Assoc 2017;117(7):451-456.
12. Bahner DP, Adkins EJ, Hughes D, et al. Integrated medical school ultrasound: development of an ultrasound vertical curriculum. Crit Ultrasound J 2013;5:6-14.
13. Wilson SP, Mefford JM, Lahham S, et al. Implementation of a 4-year point-of-care ultrasound curriculum in a liaison committee on medical education-accredited US medical school. J Ultrasound Med 20217;36:321-325.
    1. **The curriculum forms the foundation for ultrasound as a core clinical competency for all graduates, regardless of specialty choice**

Over the past two decades the health professions have been working toward competency-based medical education (CBME) models to ensure that quality care is being delivered to patients by all practitioners. CBME can be defined as an outcomes approach to the design, implementation, assessment, and evaluation of medical education programs based on a framework of competencies. Competency can be defined as an observable, measurable, and assessable ability of a health professional that includes multiple components such as the appropriate knowledge, skills, values, and attitudes for a specific competency. Competencies can be broken down into milestones which are observable steps that can be used to assess and document a learner’s progress toward a given competency along a developmental continuum (1,2)

The result of this early work on competencies has been the development of a Reference List of General Physician Competencies consisting of 8 competence domains and 58 competencies. Domains of competence are broad but distinguishable areas of competence that in the aggregate constitute a general descriptive framework for a profession. The eight identified domains for the health professions are patient care, knowledge of practice, practice-based learning and improvement, interpersonal and communication skills, professionalism, systems- based practice, interprofessional collaboration, and personal and professional development (3).

This list of competencies was then used in developing 13 core Entrustable Professional Activities (EPAs) for entering residency endorsed by the Association of American Medical Colleges. EPAs are units of professional practice, defined as tasks or responsibilities that trainees are entrusted to perform unsupervised once they have attained sufficient specific competence. EPAs are independently executable, observable, and measurable in their process and outcome (4). EPAs should also be grounded in day-to-day practice and be suitable for focused entrustable decisions (5).

Based on these criteria, ultrasound is well suited to serve as a competency component for a number of the core EPAs as it is observable, measureable, is becoming common in the day-to-day clinical practice setting, and is amenable to entrustable decisions like other medical student skills.

**Ultrasound and EPAs**

Ultrasound can be considered an important direct competency component in 5 of the 13 core EPAs:

EPA 1: Gather a history and perform a physical examination. Ultrasound has been shown with medical students to enhance and even outperform a variety of components of the traditional physical examination, such as detecting cardiac pathology, determining liver size, detecting pleural effusions, and detecting abdominal aortic aneurysms (6-9).

EPA 2: Prioritize a differential diagnosis following a clinical encounter. Ultrasound protocols have been developed that help form, prioritize, and assess the differential diagnosis for a number of common clinical scenarios like hypotension/shock (RUSH Protocol) and dyspnea (BLUE Protocol) (10-11).

EPA 3: Recommend and interpret common diagnostic and screening tests due to its safety profile, cost effectiveness, and clinical value, ultrasound is becoming a first-line diagnostic and screening test for many medical conditions across many specialties and subspecialties (12-13).

EPA 10: Recognize a patient requiring urgent or emergent care and initiate evaluation and management.

For over two decades of Emergency Medicine experience and more recently Hospitalist and Primary Care Medicine experience, ultrasound has proven to be an important first-line diagnostic and management tool for many of the urgent/emergent conditions listed in this EPA such as chest pain, shortness of breath, fever, hypertension/hypotension, tachycardia/arrhythmia, and oliguria/anuria/urinary retention (14-15).

EPA 12: Perform general procedures of a physician. Ultrasound can be considered a procedure in itself that is becoming common to the practice of many medical specialties, including all the primary care specialties. In addition, ultrasound-guidance enhances the accuracy and safety of many common procedures including two of those specifically listed in EPA 12: venipuncture and intravenous line placement. Ultrasound-guidance will likely become the standard of care for many procedures as it has become for central-line placement. These procedures include thoracentesis, paracentesis, lumbar puncture, arthrocentesis, incision and drainage of abscesses, other procedures (13).

In addition to these direct roles that ultrasound can play in these five EPAs, it can play important indirect roles in several other core EPAs such as being more knowledgeable about ordering imaging studies (EPA 4), forming clinical questions (EPA 7), collaborating on an inter-professional team (EPA 9), understanding informed consent (EPA 11), and contributing to a culture of safety and improvement (EPA 13).

**Ultrasound and Patient Safety**

Patient safety has been proclaimed as the “the primary motivation for the work on EPA’s” (4). Ultrasound does not use ionizing radiation for its imaging and therefore has a much preferred safety profile than other common imaging modalities like X-ray and computer tomography. In addition, the Agency for Healthcare Research and Quality (AHRQ) has identified the use of real-time ultrasound guidance during central line insertion as a top ten patient safety practice. The AHRQ also recommends that providers not delay adopting this practice of using ultrasound guidance (16).

**Ultrasound and Medical School Accreditation Standards**

As with EPAs, ultrasound is consistent with and contributes to several of the Liaison Committee on Medical Education standards and elements for MD education accreditation (17).

Element 6.1: The education program must be outcome based and measurement of progress in developing competencies the profession and public expect of physicians.

Ultrasound education is consistent with an outcomes-based and progressive measurement of competency model. In addition, it forms the early educational experience for the ultrasound education continuum from medical school to residency to continuing medical education. Some residencies have already established ultrasound as an official milestone such as emergency medicine and other are developing ultrasound curricula and milestones such as family medicine and general surgery (18-20).

Standard 7: Curricular content must be of sufficient breadth and depth to prepare students for any residency and the subsequent contemporary practice of medicine.

There are now clinical ultrasound applications for almost every specialty and subspecialty. Many of these ultrasound applications such as basic echocardiography cross many residency and practice specialties needs (13).

Element 8.1: One of the responsibilities of the institute is oversight of the education program including integration, coordination, and coherence of the curriculum.

As an educational tool ultrasound can effectively integrate the medical student curriculum, bridge basic and clinical sciences, and progress from a learning tool to a highly effective clinical tool as has been shown by several undergraduate integrated ultrasound curricula (21-26).

**References**

1. Frank JR, Snell LS, Cate OT, et al. Competency-based medical education: theory to practice. *Med Teach* 2010; 32:638-645.
2. Holmboe ES, Edgar L, Hamstra S. ACGME: The Milestones Guidebook. Version 2016. ACGME website: <https://www.acgme.org/Portals/0/MilestonesGuidebook.pdf>
3. Englander E, Cameron T, Ballard AJ, Dodge J, Bull J, Aschenbrener CA. Toward a common taxonomy of competency domains for the health professions and competencies for physicians. Academic Medicine 2013;88:1088-1092.
4. Core Entrustable Professional Activities for Entering Residency: Curriculum Developers’ Guide. AAMC website: <https://members.aamc.org/eweb/upload/Core%20EPA%20Curriculum%20Dev%20Guide.pdf>
5. Cate OT, Chen HC, Hoff RG, et al. Curriculum development for the workplace using entrustable professional activities (EPAs): AMEE Guide No. 99. Med Teach 2015;37:983-1002
6. Kobal SL, Trento L, Baharami S, Tolstrup K, Naqvi TZ, Cercek B, Neuman Y, Mirocha J, Kar S, Forrester JS, Siegel RJ (2005) Comparison of effectiveness of hand-carried ultrasound to bedside cardiovascular physical examination. Am J Cardiol 96:1002-1006.
7. [Mouratev G, Howe D, Hoppmann R, Poston MB, Reid R, Varnadoe J, Smith S, McCallum B, Rao V, DeMarco P (2013) Teaching medical students ultrasound to measure liver size: comparison with experienced clinicians using physical examination alone. Teach Learn Med. 25(1):84-8.](http://uscmedultrasound.pbworks.com/w/file/fetch/86695900/Teaching%20Medical%20Students%20Ultrasound%20to%20Measure%20Liver%20Size.pdf)
8. Steinmetz P, Oleskevich S, Dyachenko A, McCusker J, Lewis J.(2018) [Accuracy of Medical Students in Detecting Pleural Effusion Using Lung Ultrasound as an Adjunct to the Physical Examination.](https://www.ncbi.nlm.nih.gov/pubmed/29574857) J Ultrasound Med. 2018 Nov;37(11):2545-2552.
9. Mai T, Woo MY, Boles K, Jetty P.(2018) [Point-of-Care Ultrasound Performed by a Medical Student Compared to Physical Examination by Vascular Surgeons in the Detection of Abdominal Aortic Aneurysms.](https://www.ncbi.nlm.nih.gov/pubmed/29777851)Ann Vasc Surg. 2018 Oct;52:15-21.
10. Perera P, Mailhot T, Riley D, Mandavia D; The RUSH exam: rapid ultrasound in shock in the critically ill. Emerg Med Clin N Am, 2010;28:29-56.
11. Lichtenstein DA, Meziere GA; Relevance of lung ultrasound in the diagnosis of acute respiratory failure: the BLUE protocol. Chest, 2008;134:117-125.
12. Minton KK, Abuhamad A. [2012 Ultrasound First Forum proceedings.](https://www.ncbi.nlm.nih.gov/pubmed/23525380) J Ultrasound Med. 2013 Apr;32(4):555-66.
13. Moore, CL, Copel JA. Point-of-care ultrasonography. N Engl J Med, 2011;364:749-757.
14. Ultrasound Guidelines: Emergency, Point-of-Care and Clinical Ultrasound Guidelines in Medicine. [Ann Emerg Med.](https://www.ncbi.nlm.nih.gov/pubmed/28442101) 2017 May;69(5):e27-e54.
15. Bhagra A, Tierney DM, Sekiguchi H, Soni NJ. [Point-of-Care Ultrasonography for Primary Care Physicians and General Internists.](https://www.ncbi.nlm.nih.gov/pubmed/27825617)Mayo Clin Proc. 2016 Dec;91(12):1811-1827.
16. Agency for Healthcare Research and Quality (AHRQ) Making Healthcare Safer II:

An Updated Critical Analysis of the Evidence for Patient Safety Practices – Evidence

Report/Technology Assessment Number 211; Publication No. 13-E001-EF, March 2013

1. Liaison Committee on Medical Education (LCME). 2017. Functions and structure of a medical school: Standards for accreditation of medical education programs leading to the MD Degree. Washington, DC. USA. <http://lcme.org/publications/>
2. Accreditation Council for Graduate Medical Education (ACGME) and The American Board of Emergency Medicine. The Emergency Medical Services Milestone Project July 2015:

<https://www.acgme.org/Portals/0/PDFs/Milestones/EmergencyMedicalServicesMilestones.pdf?ver=2015-11-06-120532-130>

1. Bornemann . Assessment of a novel point-of-care ultrasound curriculum’s effect on competency measures in family medicine graduate medical education. J Ultrasound Med. 2017;36:1205-1211.
2. Beal E, Sigmond b, Sage-Silski L, et al. Point-of-care ultrasound in general surgery residency training: a proposal for milestones in graduate medical education ultrasound. J Ultrasound Med. 2017;36:2577-2584.
3. Hoppmann R, Rao V, Bell F, et al. The evolution of an integrated ultrasound curriculum (iUSC) for medical students: 9-year experience. Crit Ultrasound J. 2015;7(1):18-33.
4. Palma JK. Successful strategies for integrating bedside ultrasound into undergraduate medical education. Military Medicine 2015;4:153-157.
5. Rao S, van Holsbeeck L, Musial JL, Parker A, Bouffard JA, Bridge P, Jackson M, Dulchavsky SA (2008) A pilot study of comprehensive ultrasound education at the Wayne State University School of Medicine: a pioneer year review. J Ultrasound Med 27(5):745-749.
6. Russ BA, Evans D, Morrad D, et al. Integrating point-of-care ultrasonography into the osteopathic medical school curriculum. J AM Osteop Assoc 2017;117(7):451-456.
7. Bahner DP, Adkins EJ, Hughes D, et al. Integrated medical school ultrasound: development of an ultrasound vertical curriculum. Crit Ultrasound J 2013;5:6-14.
8. Wilson SP, Mefford JM, Lahham S, et al. Implementation of a 4-year point –of-care ultrasound curriculum in a liaison committee on medical education-accredited US medical school. J Ultrasound Med 20217;36:321-325.

**1.3 The curriculum provides the foundation of ultrasound for all medical students, regardless of where their medical degree is obtained or the specific designation of their degree.**

There is significant overlap in the standards, principles, and objectives of medical student education for physicians globally regardless of the specific medical degree conferred such as Doctor of Medicine (MD), Doctor of Osteopathy (DO), and Bachelor of Medicine/Bachelor of Surgery (MBBS). These include a solid foundation in the human and biological sciences, integration of the basic and clinical sciences, a competency-based educational approach, broad clinical preparation for the practice of medicine, inter-professional practice, preparation for a continuum of learning, and patient safety (1-5).

Thus, a recommended ultrasound curriculum for those in training to become physicians should provide a foundation to support as many of these standards, principles, and objectives as possible making it universally applicable.

**References**

1. Liaison Committee on Medical Education (LCME). 2017. Functions and structure of a medical school: Standards for accreditation of medical education programs leading to the MD Degree. Washington, DC. USA. <http://lcme.org/publications/>
2. Accreditation of Colleges of Osteopathic Medicine: COM Accreditation Standards and Procedures (effective August 29, 2016). <https://osteopathic.org/wp-content/uploads/2018/02/com-accreditation-standards-8-29-2016.pdf>
3. Diretrizes Curriculares Nacionais do Curso de Graduação em Medicina (National Curricular Guidelines of the Undergraduate Medical Course): <http://www.fmb.unesp.br/Home/Graduacao/resolucao-dcn-2014.pdf>
4. General Medical Council: Standards and Outcomes <https://www.gmc-uk.org/education/standards-guidance-and-curricula/standards-and-outcomes> (website accessed 1.18.19)
5. Australian Medical Council Limited. Standards for assessment and accreditation of primary medical programs by the Australian Medical Council 2012. Kingston, ACT. Australia. <http://www.amc.org.au/files/d0ffcecda9608cf49c66c93a79a4ad549638bea0_original.pdf>

**1.4 The curriculum can serve as a valuable resource for the development of ultrasound training programs for non-physician healthcare providers such as advanced nurse practitioners and physician assistants.**

Considering the overlap in medical student educational content and skill with that of other healthcare professionals as set by their accrediting bodies such as nurse practitioners, nurses, physician assistants, and emergency medicine technicians, an integrated ultrasound curriculum for medical students should prove to be a valuable and appropriate resource for the education of these and other healthcare professionals as well (1-5).

It has been demonstrated that non-physician providers can learn and competently use ultrasound in the clinical setting (6-9).

With a common clinical skill like ultrasound, having a variety of health professionals trained with a similar basic curriculum offers an excellent opportunity for inter-professional training. Both the LCME standards (standard/element 7.9) and the AAMC core EPAs (EPA 9) emphasize the importance of collaboration on an inter-professional team (1,10).

**References**

1. Liaison Committee on Medical Education (LCME). 2017. Functions and structure of a medical school: Standards for accreditation of medical education programs leading to the MD Degree. Washington, DC. USA. <http://lcme.org/publications/>
2. Accreditation Standards of Physician Assistant Education. Fourth Edition: <http://www.arc-pa.org/wp-content/uploads/2018/12/AccredManual-4th-edition.rev5_.18.pdf>
3. Standards and Guidelines for the Accreditation of Educational Programs in the Emergency Medical Services Professions. <https://www.caahep.org/CAAHEP/media/CAAHEP-Documents/EMSPStandards2015.pdf>
4. American Nurses Association Nursing: Scope and Standards of Practice, Third Edition – 2015. <https://www.augusta.edu/nursing/cnr/documents/seminar-files/pp8.28.pdf>
5. Criteria for Evaluation of Nurse Practitioner Programs 5^th^ Edition 2016 by The National Task Force on Quality Nurse Practitioner Education. <http://www.acenursing.net/resources/NTF_EvalCriteria2016Final.pdf>
6. Atkinson P, Madan R, Kendall R, et al. Detection of soft tissue foreign bodies by nurse practitioner-performed ultrasound. Critical Ultrasound Journal 2014;6(1):2. <https://theultrasoundjournal.springeropen.com/articles/10.1186/2036-7902-6-2>
7. Mumoli N, Vitale J, Griorgi-Pierfranceschi M, et al. Accuracy of nurse-performed lung ultrasound in patients with acute dyspnea. Medicine 2016;95:1-5.
8. Balog TP, Rhodehouse BB, Turner EK, et al. Accuracy of ultrasound-guided intra-articular hip injections performed in the orthopedic clinic. Orthopedics 2017;40(2):96-100.
9. Duran-Gehring P, Bryant L, Reynolds J, et al. Ultrasound-guided peripheral intravenous catheter training results in physician-level success for emergency department technicians. J Ultrasound Med 2016;35:2343-2352.
10. Core Entrustable Professional Activities for Entering Residency: Curriculum Developers’ Guide. AAMC website: <https://members.aamc.org/eweb/upload/Core%20EPA%20Curriculum%20Dev%20Guide.pdf>

**Domain 2: Rationale for Teaching Ultrasound**

**2.1 The curriculum prepares students for POCUS (point of care ultrasound use) in future clinical work.**

Point of Care Ultrasound (PoCUS) has been successfully introduced into the curricula of many medical schools, especially in N. America – and especially in anatomy and physiology (1-4). There is a growing body of evidence that this exposure to a medical school ultrasound curriculum better prepares students for clinical ultrasound use as they progress through their education (5-8). This includes procedural guidance skills, diagnostic ultrasound and PoCUS protocols which appear to be retained when the student reaches the post-graduate residency training level (9-10).

**References**

1. Rao S, van Holsbeeck L, Musial JL, et al. A pilot study of comprehensive ultrasound education at the Wayne State University School of Medicine: a pioneer year review. J Ultrasound Med. 2008;27(5):745-749. http://www.ncbi.nlm.nih.gov/pubmed/18424650. Accessed January 30, 2018.

2. Bahner DP, Adkins EJ, Hughes D, Barrie M, Boulger CT, Royall NA. Integrated medical school ultrasound: development of an ultrasound vertical curriculum. Crit Ultrasound J. 2013;5(1):6. doi:10.1186/2036-7902-5-6.

3. Soucy ZP, Mills LD. American Academy of Emergency Medicine Position Statement: Ultrasound Should Be Integrated into Undergraduate Medical Education Curriculum. J Emerg Med. 2015;49(1):89-90. doi:10.1016/j.jemermed.2014.12.092.

4. Bahner DP, Royall NA. Advanced Ultrasound Training for Fourth-Year Medical Students. Acad Med. 2013;88(2):206-213. doi:10.1097/ACM.0b013e31827c562d.

5. Steller J, Russell B, Lotfipour S, et al. USEFUL: Ultrasound Exam for Underlying Lesions Incorporated into Physical Exam. West J Emerg Med. 2014;15(3):260-266. doi:10.5811/westjem.2013.8.19080. 16

6. Eissa K, Alokla K, Assasa O, Itani A, Shawwa K, Kheir F. Using Simulation Based Training to Incorporate Thoracic Ultrasound into Physical Examination. J La State Med Soc. 167(6):264-267. http://www.ncbi.nlm.nih.gov/pubmed/26741686. Accessed April 26, 2017.

7. Arger PH, Schultz SM, Sehgal CM, Cary TW, Aronchick J. Teaching medical students diagnostic sonography. J Ultrasound Med. 2005;24(10):1365-1369. http://www.ncbi.nlm.nih.gov/pubmed/16179619. Accessed January 30, 2018.

8. Fox JC, Cusick S, Scruggs W, et al. Educational assessment of medical student rotation in emergency ultrasound. West J Emerg Med. 2007;8(3):84-87. http://www.ncbi.nlm.nih.gov/pubmed/19561689. Accessed January 30, 2018.
9. Wilson SP, Mefford JM, Lahham S, et al. Implementation of a 4-Year Point-of-Care Ultrasound Curriculum in a Liaison Committee on Medical Education Accredited US Medical School. J Ultrasound Med. 2017;36(2):321-325. doi:10.7863/ultra.16.03068.

10. Udrea DS, Sumnicht A, Lo D, et al. Effects of Student-Performed Point-of-Care Ultrasound on Physician Diagnosis and Management of Patients in the Emergency Department. J Emerg Med. 2017;53(1):102-109. doi:10.1016/j.jemermed.2017.01.021.

**2.2 The curriculum facilitates teaching of fundamental sciences.**

The use of ultrasound in undergraduate medical education began as a way to augment the teaching of anatomy and physiology. Here, the evidence for improving a student’s understanding has been positive, both in terms of student engagement and in improving knowledge and confidence -- indeed comparable to cadaveric anatomic and physiologic knowledge acquisition (1-4). There is some evidence that ultrasound guided anatomy also improves test scores (5-8).

**References**

1. Swamy M, Searle RF. Anatomy teaching with portable ultrasound to medical students. BMC Med Educ. 2012;12(1):99. doi:10.1186/1472-6920-12-99.

2. Tshibwabwa ET, Groves HM. Integration of ultrasound in the education programme in anatomy. Med Educ. 2005;39(11):1148-1148. doi:10.1111/j.1365- 2929.2005.02288.x.

3. Stringer MD, Duncan LJ, Samalia L. Using real-time ultrasound to teach living anatomy: an alternative model for large classes. N Z Med J. 2012;125(1361):37- 45. http://www.ncbi.nlm.nih.gov/pubmed/22960714. Accessed January 30, 2018.

4. Griksaitis MJ, Sawdon MA, Finn GM. Ultrasound and cadaveric prosections as methods for teaching cardiac anatomy: A comparative study. Anat Sci Educ. 2012;5(1):20-26. doi:10.1002/ase.259.

5. Kondrashov P, Johnson JC, Boehm K, Rice D, Kondrashova T. Impact of the clinical ultrasound elective course on retention of anatomical knowledge by second-year medical students in preparation for board exams. Clin Anat. 2015;28(2):156-163. doi:10.1002/ca.22494.

6. Paganini M, Bondì M, Rubini A. Evaluation of chest ultrasound integrated teaching of respiratory system physiology to medical students. Adv Physiol Educ. 2017;41(4):514-517. doi:10.1152/advan.00062.2017. 18

7. Feilchenfeld Z, Dornan T, Whitehead C, Kuper A. Ultrasound in undergraduate medical education: a systematic and critical review. Med Educ. 2017;51(4):366- 378. doi:10.1111/medu.13211.

8. Serrao G, Tassoni M, Magenta-Biasina AM, et al. Competency-based medical education studying live anatomy by ultrasound. Int J Med Educ. 2017;8:268-269. doi:10.5116/ijme.595f.b183.

**2.3 The curriculum enhances the learning of clinical sciences.**

There is limited evidence supporting the implementation of a medical school ultrasound curriculum impacting the clinical practice of medicine. There is less research on the translation of basic ultrasound anatomy and physiology understanding into the performance of students during their clinical rotations, their understanding of pathology, and their management of diseases (1-2)

**References**

1. Wright SA, Bell AL. Enhancement of undergraduate rheumatology teaching through the use of musculoskeletal ultrasound. Rheumatology (Oxford). 2008;47(10):1564-1566. doi:10.1093/rheumatology/ken324.

2. Alfageme F, Cerezo E, Fernandez I, Aguilo R, Vilas-Sueiro A, Roustan G. Introduction of Basic Dermatologic Ultrasound in Undergraduate Medical Education. Ultrasound Int Open. 2016;02(04):E136-E139. doi:10.1055/s-0042- 120273.

**2.4 The curriculum facilitates integration of basic and clinical sciences.**

Evidence in support of improved basic and clinical sciences integration through PoCUS is indirect at best. Based on the current literature, educators report increased levels of knowledge and/or diagnostic skills suggested by increased PoCUS knowledge in that anatomic area (1-3). This link is mostly suggested in fields where the clinical picture is more directly dependent upon organ-system anatomy and/or physiology (eg. cardiovascular system) (4-5). However, the integration of anatomy and clinical decision making is more apparent with the use of educational methods such as case-based learning. The contribution of ultrasound exposure in the learning of anatomy and physiology during medical school to patient management has not been well established.

**References**

1. Tshibwabwa ET, Groves HM, Levine MAH. Teaching musculoskeletal ultrasound in the undergraduate medical curriculum. Med Educ. 2007;41(5):517- 518. doi:10.1111/j.1365-2929.2007.02745.x.

2. Wright SA, Bell AL. Enhancement of undergraduate rheumatology teaching through the use of musculoskeletal ultrasound. Rheumatology (Oxford). 2008;47(10):1564-1566. doi:10.1093/rheumatology/ken324.

3. Kafer I, Rennie W, Noor A, Pellerito JS. Hunger Games: Interactive Ultrasound Imaging for Learning Gastrointestinal Physiology. J Ultrasound Med. 2017;36(2):361-365. doi:10.7863/ultra.16.01048

4. Panoulas VF, Daigeler A-L, Malaweera ASN, et al. Pocket-size hand-held cardiac ultrasound as an adjunct to clinical examination in the hands of medical students and junior doctors. Eur Heart J Cardiovasc Imaging. 2013;14(4):323-330. doi:10.1093/ehjci/jes140.

5. Komasawa N, Mihara R, Hattori K, Minami T. Evaluation of artery and vein differentiation methods using ultrasound imaging among medical students. Br J Anaesth. 2016;117(6):832-833. doi:10.1093/bja/aew371.

**2.5 The curriculum enhances physical examination skills.**

Point of Care Ultrasound (PoCUS) has the most research support in demonstrating improvement in physical exam performance and understanding. There are numerous studies that medical students can better identify signs of disease using PoCUS (1-6). Yet, there is fundamental dichotomy in this premise; does PoCUS use enhance the physical exam by virtue of better test characteristics for PoCUS vs. physical exam or does PoCUS use also improve the actual performance of the physical exam by medical students. Several landmark papers have shown PoCUS to be superior to classical cardiac physical exam findings - even when performed by experts (1).

On the other hand, there is also evidence that learning PoCUS enhances a students’ classical clinical exam skills by enabling students to better understand regional anatomy and composition (7-10).

Most studies show increased student confidence in performing the physical exam but there are limited data suggesting that this translates to improved clinical management. (11-16).

**References**

1. Kobal SL, Trento L, Baharami S, et al. Comparison of effectiveness of handcarried ultrasound to bedside cardiovascular physical examination. Am J Cardiol. 2005;96(7):1002-1006. doi:10.1016/j.amjcard.2005.05.060.

2. Shmueli H, Burstein Y, Sagy I, et al. Briefly trained medical students can effectively identify rheumatic mitral valve injury using a hand-carried ultrasound. Echocardiography. 2013;30(6):621-626. doi:10.1111/echo.12122.

3. Decara JM, Kirkpatrick JN, Spencer KT, et al. Use of hand-carried ultrasound devices to augment the accuracy of medical student bedside cardiac diagnoses. J Am Soc Echocardiogr. 2005;18(3):257-263. doi:10.1016/j.echo.2004.11.015.

4. Hoppmann R, Hunt P, Louis H, et al. Medical student identification of knee effusion by ultrasound. ISRN Rheumatol. 2011;2011:874596. doi:10.5402/2011/874596.

5. Panoulas VF, Daigeler A-L, Malaweera ASN, et al. Pocket-size hand-held cardiac ultrasound as an adjunct to clinical examination in the hands of medical 22 students and junior doctors. Eur Heart J Cardiovasc Imaging. 2013;14(4):323-330. doi:10.1093/ehjci/jes140.

6. Mouratev G, Howe D, Hoppmann R, et al. Teaching medical students ultrasound to measure liver size: comparison with experienced clinicians using physical examination alone. Teach Learn Med. 2013;25(1):84-88. doi:10.1080/10401334.2012.741535.

7. Andersen GN, Viset A, Mjølstad OC, Salvesen Ø, Dalen H, Haugen BO. Feasibility and accuracy of point-of-care pocket-size ultrasonography performed by medical students. BMC Med Educ. 2014;14(1):156. doi:10.1186/1472-6920-14- 156.

8. Stokke TM, Ruddox V, Sarvari SI, Otterstad JE, Aune E, Edvardsen T. Brief Group Training of Medical Students in Focused Cardiac Ultrasound May Improve Diagnostic Accuracy of Physical Examination. J Am Soc Echocardiogr. 2014;27(11):1238-1246. doi:10.1016/j.echo.2014.08.001.

9. Ruiz-Curiel A, Díaz-Barreda MD, González-Rodríguez M, et al. Musculoskeletal ultrasound: an effective tool to help medical students improve joint inflammation detection? Med Ultrason. 2016;18(3):294. doi:10.11152/mu.2013.2066.183.ruz.

10. Walrod BJ, Schroeder A, Conroy MJ, et al. Does Ultrasound-Enhanced Instruction of Musculoskeletal Anatomy Improve Physical Examination Skills of First-Year Medical Students? J Ultrasound Med. 2018;37(1):225-232. doi:10.1002/jum.14322.

11. Feilchenfeld Z, Dornan T, Whitehead C, Kuper A. Ultrasound in undergraduate medical education: a systematic and critical review. Med Educ. 2017;51(4):366-378. doi:10.1111/medu.13211.

12. Butter J, Grant TH, Egan M, et al. Does ultrasound training boost Year 1 medical student competence and confidence when learning abdominal examination? Med Educ. 2007;41(9):843-848. doi:10.1111/j.1365- 2923.2007.02848.x.

13. de Vries KD, Brown R, Mazzie J, Jung M-K, Yao SC, Terzella MJ. Effect of Ultrasonography on Student Learning of Shoulder Anatomy and Landmarks. J Am Osteopath Assoc. 2018;118(1):34. doi:10.7556/jaoa.2018.006.

14. Woods R, Wisniewski SJ, Lueders DR, Pittelkow TP, Larson DR, Finnoff JT. Can Ultrasound Be Used to Improve the Palpation Skills of Physicians in Training? A Prospective Study. PM&R. December 2017. doi:10.1016/j.pmrj.2017.11.016.

15. Parikh T, Czuzak M, Bui N, et al. Novel Use of Ultrasound to Teach Reproductive System Physical Examination Skills and Pelvic Anatomy. J Ultrasound Med. 2017;37(3):709-715. doi:10.1002/jum.14408.

16. Ahn JS, French AJ, Thiessen MEW, et al. Using Ultrasound to Enhance Medical Students’ Femoral Vascular Physical Examination Skills. J Ultrasound Med. 2015;34(10):1771-1776. doi:10.7863/ultra.15.14.11014.

**2.6 The curriculum enhances clinical problem solving.**

There is some evidence that students can master the skill of obtaining PoCUS images and correctly interpret these to attain the correct diagnoses (1). In addition, medical student PoCUS images have on occasion demonstrated new findings that alter diagnostic decision making and reduce time to disposition (2-3).

**References**

1. Udrea DS, Sumnicht A, Lo D, et al. Effects of Student-Performed Point-of-Care Ultrasound on Physician Diagnosis and Management of Patients in the Emergency Department. J Emerg Med. 2017;53(1):102-109. doi:10.1016/j.jemermed.2017.01.021.)

2. Amini R, Stolz L, Hernandez N, et al. Sonography and hypotension: a change to critical problem solving in undergraduate medical education. Adv Med Educ Pract. 2016;7:7. doi:10.2147/AMEP.S97491.

3. Parks AR, Verheul G, LeBlanc-Duchin D, Atkinson P. Effect of a point-of-care ultrasound protocol on the diagnostic performance of medical learners during simulated cardiorespiratory scenarios. CJEM. 2015;17(03):263-269. doi:10.1017/cem.2014.41.

**2.7 The curriculum prepares learners for additional clinical training and/or practice opportunities.**

Several articles have suggested that exposure to medical school ultrasound curricula may alter students’ decisions on the residency they choose and on activities they participate in - like increased participation in peer-to-peer teaching (1-16).

**References**

1. Fox JC, Lahham S, Maldonado G, et al. Hypertrophic Cardiomyopathy in Youth Athletes: Successful Screening With Point-of-Care Ultrasound by Medical Students. J Ultrasound Med. 2017;36(6):1109-1115. doi:10.7863/ultra.16.06044.

2. Bahner DP, Jasne A, Boore S, Mueller A, Cortez E. The ultrasound challenge: a novel approach to medical student ultrasound education. J Ultrasound Med. 2012;31(12):2013-2016. http://www.ncbi.nlm.nih.gov/pubmed/23197555. Accessed January 30, 2018.

3. Udrea DS, Sumnicht A, Lo D, et al. Effects of Student-Performed Point-of-Care Ultrasound on Physician Diagnosis and Management of Patients in the Emergency Department. J Emerg Med. 2017;53(1):102-109. doi:10.1016/j.jemermed.2017.01.021.

4. Barsky M, Kushner L, Ansbro M, et al. A feasibility study to determine if minimally trained medical students can identify markers of chronic parasitic infection using bedside ultrasound in rural Tanzania. World J Emerg Med. 2015;6(4):293. doi:10.5847/wjem.j.1920-8642.2015.04.008.

5. Lee JB, Tse C, Keown T, et al. Evaluation of a point of care ultrasound curriculum for Indonesian physicians taught by first-year medical students. World J Emerg Med. 2017;8(4):281. doi:10.5847/wjem.j.1920-8642.2017.04.006.

6. Heinzow HS, Friederichs H, Lenz P, et al. Teaching ultrasound in a curricular course according to certified EFSUMB standards during undergraduate medical education: a prospective study. BMC Med Educ. 2013;13(1):84. doi:10.1186/1472- 6920-13-84. 26

7. Steller J, Russell B, Lotfipour S, et al. USEFUL: Ultrasound Exam for Underlying Lesions Incorporated into Physical Exam. West J Emerg Med. 2014;15(3):260-266. doi:10.5811/westjem.2013.8.19080.

8. Prats MI, Royall NA, Panchal AR, Way DP, Bahner DP. Outcomes of an Advanced Ultrasound Elective. J Ultrasound Med. 2016;35(5):975-982. doi:10.7863/ultra.15.06060.

9. Kondrashova T, Lockwood MD. Innovative Approach to Teaching Osteopathic Manipulative Medicine: The Integration of Ultrasonography. J Am Osteopath Assoc. 2015;115(4):212. doi:10.7556/jaoa.2015.043.

10. Tokumine J, Matsushima H, Lefor AK, Igarashi H, Ono K. Ultrasound-guided subclavian venipuncture is more rapidly learned than the anatomic landmark technique in simulation training. J Vasc Access. 2015;16(2):144-147. doi:10.5301/jva.5000318.

11. Blackstock U, Munson J, Szyld D. Bedside ultrasound curriculum for medical students: Report of a blended learning curriculum implementation and validation. J Clin Ultrasound. 2015;43(3):139-144. doi:10.1002/jcu.22224.

12. Naeger DM, Conrad M, Nguyen J, Kohi MP, Webb EM. Students Teaching Students. Acad Radiol. 2013;20(9):1177-1182. doi:10.1016/j.acra.2013.04.004.

13. Ahn JS, French AJ, Thiessen MEW, Kendall JL. Training Peer Instructors for a Combined Ultrasound/Physical Exam Curriculum. Teach Learn Med. 2014;26(3):292-295. doi:10.1080/10401334.2014.910464.

14. Shapiro RS, Ko PK, Jacobson S. A pilot project to study the use of ultrasonography for teaching physical examination to medical students. Comput Biol Med. 2002;32(6):403-409. http://www.ncbi.nlm.nih.gov/pubmed/12356490. Accessed January 30, 2018.(“equired.”)

15. Dickerson J, Paul K, Vila P, Whiticar R. The role for peer-assisted ultrasound teaching in medical school. Clin Teach. 2017;14(3):170-174. doi:10.1111/tct.12541.

16. Denny SP, Minteer WB, Fenning RTH, et al. Ultrasound curriculum taught by first-year medical students: A four-year experience in Tanzania. World J Emerg Med. 2018;9(1):33. doi:10.5847/wjem.j.1920-8642.2018.01.005.

**2.8 The curriculum enhances the overall educational experience***.*

Early PoCUS research on medical student exposure has focused on student satisfaction. We have yet to find a report where students did not find PoCUS utilization in teaching enjoyable (1-8). In contrast, there is some evidence that students can be over-confident in their PoCUS skills or image interpretation at a time when they have limited understanding of the underlying core principles of patient management (9-10). Authors thus suggest that PoCUS might be better incorporated as a supplemental skill. PoCUS is described as motivating students to delve deeper into matters of interest. It does not appear to adversely impact the time needed to teach other content during an already over-crowded undergraduate curriculum (11-12).

**References**

1. Wright SA, Bell AL. Enhancement of undergraduate rheumatology teaching through the use of musculoskeletal ultrasound. Rheumatology (Oxford). 2008;47(10):1564-1566. doi:10.1093/rheumatology/ken324.

2. Eissa K, Alokla K, Assasa O, Itani A, Shawwa K, Kheir F. Using SimulationBased Training to Incorporate Thoracic Ultrasound into Physical Examination. J La State Med Soc. 167(6):264-267. http://www.ncbi.nlm.nih.gov/pubmed/26741686. Accessed April 26, 2017.

3. Tshibwabwa ET, Groves HM, Levine MAH. Teaching musculoskeletal ultrasound in the undergraduate medical curriculum. Med Educ. 2007;41(5):517- 518. doi:10.1111/j.1365-2929.2007.02745.x.

4. Hammoudi N, Arangalage D, Boubrit L, et al. Ultrasound-based teaching of cardiac anatomy and physiology to undergraduate medical students. Arch Cardiovasc Dis. 2013;106(10):487-491. doi:10.1016/j.acvd.2013.06.002.

5. Webb EM, Cotton JB, Kane K, Straus CM, Topp KS, Naeger DM. Teaching Point of Care Ultrasound Skills in Medical School. Acad Radiol. 2014;21(7):893- 901. doi:10.1016/j.acra.2014.03.001. 28

6. Hammoudi N, Arangalage D, Boubrit L, et al. Ultrasound-based teaching of cardiac anatomy and physiology to undergraduate medical students. Arch Cardiovasc Dis. 2013;106(10):487-491. doi:10.1016/j.acvd.2013.06.002.

7. Bell FE, Wilson LB, Hoppmann RA. Using ultrasound to teach medical students cardiac physiology. Adv Physiol Educ. 2015;39(4):392-396. doi:10.1152/advan.00123.2015.

8. Favot M, Courage C, Mantouffel J, Amponsah D. Ultrasound Training in the Emergency Medicine Clerkship. West J Emerg Med. 2015;16(6):938-942. doi:10.5811/westjem.2015.9.27290.

9. Sweetman GM, Crawford G, Hird K, Fear MW. The benefits and limitations of using ultrasonography to supplement anatomical understanding. Anat Sci Educ. 2013;6(3):141-148. doi:10.1002/ase.1327.

10. Hoppmann RA, Rao V V., Bell F, et al. The evolution of an integrated ultrasound curriculum (iUSC) for medical students: 9-year experience. Crit Ultrasound J. 2015;7(1):18. doi:10.1186/s13089-015-0035-3

11. Rao S, van Holsbeeck L, Musial JL, et al. A pilot study of comprehensive ultrasound education at the Wayne State University School of Medicine: a pioneer year review. J Ultrasound Med. 2008;27(5):745-749. http://www.ncbi.nlm.nih.gov/pubmed/18424650. Accessed January 30, 2018.

12. Fox JC, Schlang JR, Maldonado G, Lotfipour S, Clayman R V. Proactive medicine: the “UCI 30,” an ultrasound-based clinical initiative from the University of California, Irvine. Acad Med. 2014;89(7):984-989. doi:10.1097/ACM.0000000000000292.

**2.9 Medical students can learn basic ultrasound.**

There is ample evidence that students can learn basic ultrasound and ultrasound applications, including both image acquisition and image interpretation (1-26). Image integration still requires a degree of clinical knowledge that exposure to ultrasound anatomy and physiology alone does not confer. Research has established rough estimates of learning curves that are likely attainable by the majority of medical students (27).

**References**

1. Rao S, van Holsbeeck L, Musial JL, et al. A pilot study of comprehensive ultrasound education at the Wayne State University School of Medicine: a pioneer year review. J Ultrasound Med. 2008;27(5):745-749. http://www.ncbi.nlm.nih.gov/pubmed/18424650. Accessed January 30, 2018.

2. Brascher A-K, Blunk JA, Bauer K, Feldmann R, Benrath J. Comprehensive curriculum for phantom-based training of ultrasound-guided intercostal nerve and stellate ganglion blocks. Pain Med. 2014;15(10):1647-1656. doi:10.1111/pme.12365.

3. Hoppmann RA, Rao V V, Poston MB, et al. An integrated ultrasound curriculum (iUSC) for medical students: 4-year experience. Crit Ultrasound J. 2011;3(1):1-12. doi:10.1007/s13089-011-0052-9.

4. Wong I, Jayatilleke T, Kendall R, Atkinson P. Feasibility of a focused ultrasound training programme for medical undergraduate students. Clin Teach. 2011;8(1):3-7. doi:10.1111/j.1743-498X.2010.00416.x.

5. Cawthorn TR, Nickel C, O’Reilly M, et al. Development and evaluation of methodologies for teaching focused cardiac ultrasound skills to medical students. J Am Soc Echocardiogr. 2014;27(3):302-309. doi:10.1016/j.echo.2013.12.006.

6. Hope MD, de la Pena E, Yang PC, Liang DH, McConnell M V, Rosenthal DN. A visual approach for the accurate determination of echocardiographic left ventricular ejection fraction by medical students. J Am Soc Echocardiogr. 2003;16(8):824-831. doi:10.1067/S0894-7317(03)00400-0.

7. Galusko V, Khanji MY, Bodger O, Weston C, Chambers J, Ionescu A. Handheld Ultrasound Scanners in Medical Education: A Systematic Review. J Cardiovasc Ultrasound. 2017;25(3):75. doi:10.4250/jcu.2017.25.3.75.

8. Dinh VA, Dukes WS, Prigge J, Avila M. Ultrasound Integration in Undergraduate Medical Education: Comparison of Ultrasound Proficiency Between Trained and Untrained Medical Students. J Ultrasound Med. 2015;34(10):1819-1824. doi:10.7863/ultra.14.12045.

9. Mandavia DP, Aragona J, Chan L, Chan D, Henderson SO. Ultrasound training for emergency physicians--a prospective study. Acad Emerg Med. 2000;7(9):1008- 1014. http://www.ncbi.nlm.nih.gov/pubmed/11043996. Accessed January 30, 2018.

10. Gogalniceanu P, Sheena Y, Kashef E, Purkayastha S, Darzi A, Paraskeva P. Is basic emergency ultrasound training feasible as part of standard undergraduate medical education? J Surg Educ. 2010;67(3):152-156. doi:10.1016/j.jsurg.2010.02.008.

11. Eissa K, Alokla K, Assasa O, Itani A, Shawwa K, Kheir F. Using Simulation Based Training to Incorporate Thoracic Ultrasound into Physical Examination. J La State Med Soc. 167(6):264-267. http://www.ncbi.nlm.nih.gov/pubmed/26741686. Accessed April 26, 2017.

12. Yoo MC, Villegas L, Jones DB. Basic ultrasound curriculum for medical students: validation of content and phantom. J Laparoendosc Adv Surg Tech A. 2004;14(6):374-379. doi:10.1089/lap.2004.14.374.

13. Arger PH, Schultz SM, Sehgal CM, Cary TW, Aronchick J. Teaching medical students diagnostic sonography. J Ultrasound Med. 2005;24(10):1365-1369. http://www.ncbi.nlm.nih.gov/pubmed/16179619. Accessed January 30, 2018.

14. Tshibwabwa ET, Groves HM. Integration of ultrasound in the education programme in anatomy. Med Educ. 2005;39(11):1148-1148. doi:10.1111/j.1365- 2929.2005.02288.x.

15. Nguyen HB, Losey T, Rasmussen J, et al. Interrater reliability of cardiac output measurements by transcutaneous Doppler ultrasound: implications for noninvasive hemodynamic monitoring in the ED. Am J Emerg Med. 2006;24(7):828-835. doi:10.1016/j.ajem.2006.05.012.

16. Hoppmann R, Cook T, Hunt P, et al. Ultrasound in medical education: a vertical curriculum at the University of South Carolina School of Medicine. J S C Med Assoc. 2006;102(10):330-334. http://www.ncbi.nlm.nih.gov/pubmed/17703842. Accessed January 30, 2018. 31

17. Angtuaco TL, Hopkins RH, DuBose TJ, Bursac Z, Angtuaco MJ, Ferris EJ. Sonographic physical diagnosis 101: teaching senior medical students basic ultrasound scanning skills using a compact ultrasound system. Ultrasound Q. 2007;23(2):157-160. doi:10.1097/01.ruq.0000263847.00185.28.

18. Afonso N, Amponsah D, Yang J, et al. Adding new tools to the black bag-- introduction of ultrasound into the physical diagnosis course. J Gen Intern Med. 2010;25(11):1248-1252. doi:10.1007/s11606-010-1451-5.

19. Cheng W-C, Lin X-Z, Chen C-Y. Using modern teaching strategies to teach upper abdominal sonography to medical students. J Chin Med Assoc. 2013;76(7):395-400. doi:10.1016/j.jcma.2013.03.011.

20. Oveland NP, Lossius HM, Aagaard R, Connolly J, Sloth E, Knudsen L. Animal laboratory training improves lung ultrasound proficiency and speed. J Emerg Med. 2013;45(3):e71-8. doi:10.1016/j.jemermed.2013.03.029.

21. Krause C, Krause R, Krause R, Gomez N, Jafry Z, Dinh VA. Effectiveness of a 1-Hour Extended Focused Assessment With Sonography in Trauma Session in the Medical Student Surgery Clerkship. J Surg Educ. 2017;74(6):968-974. doi:10.1016/j.jsurg.2017.03.007.

22. Kobal SL, Lior Y, Ben-Sasson A, Liel-Cohen N, Galante O, Fuchs L. The feasibility and efficacy of implementing a focused cardiac ultrasound course into a medical school curriculum. BMC Med Educ. 2017;17(1):94. doi:10.1186/s12909- 017-0928-x.

23. Syperda VA, Trivedi PN, Melo LC, et al. Ultrasonography in preclinical education: a pilot study. J Am Osteopath Assoc. 2008;108(10):601-605. http://www.ncbi.nlm.nih.gov/pubmed/18948643. Accessed January 30, 2018.

24. Fox JC, Cusick S, Scruggs W, et al. Educational assessment of medical student rotation in emergency ultrasound. West J Emerg Med. 2007;8(3):84-87. http://www.ncbi.nlm.nih.gov/pubmed/19561689. Accessed January 30, 2018.

25. Fernández-Frackelton M, Peterson M, Lewis RJ, Pérez JE, Coates WC. A bedside ultrasound curriculum for medical students: prospective evaluation of skill acquisition. Teach Learn Med. 2007;19(1):14-19. doi:10.1080/10401330709336618.

26. Ang J, Doyle B, Allen P, Cheek C. Teaching bedside ultrasound to medical students. Clin Teach. August 2017. doi:10.1111/tct.12692.

27. Kim S-C, Hauser S, Staniek A, Weber S. Learning curve of medical students in ultrasound-guided simulated nerve block. J Anesth. 2014;28(1):76-80. doi:10.1007/s00540-013-1680-y

**2.10 Medical students can learn ultrasound-guided procedures. Very strong recommendation?**

The majority of evidence for this statement comes from the ample documentation on successfully teaching students to perform venous cannulation (peripheral or central) under ultrasound guidance. However, other procedures have literature support as well (1-10).

**References**

1. Griswold-Theodorson S, Hannan H, Handly N, et al. Improving patient safety with ultrasonography guidance during internal jugular central venous catheter placement by novice practitioners. Simul Healthc. 2009;4(4):212-216. doi:10.1097/SIH.0b013e3181b1b837.

2. Moak JH, Rajkumar JS, Woods WA. The wire is really easy to see (WIRES): sonographic visualization of the guidewire by novices. CJEM. 2013;15(1):18-23. http://www.ncbi.nlm.nih.gov/pubmed/23283119. Accessed January 30, 2018.

3. Kim S-C, Hauser S, Staniek A, Weber S. Learning curve of medical students in ultrasound-guided simulated nerve block. J Anesth. 2014;28(1):76-80. doi:10.1007/s00540-013-1680-y.

4. Breslin R, Collins K, Cupitt J. The use of ultrasound as an adjunct to peripheral venous cannulation by junior doctors in clinical practice. Med Teach. January 2018:1-6. doi:10.1080/0142159X.2018.1428737.

5. Brascher A-K, Blunk JA, Bauer K, Feldmann R, Benrath J. Comprehensive curriculum for phantom-based training of ultrasound-guided intercostal nerve and stellate ganglion blocks. Pain Med. 2014;15(10):1647-1656. doi:10.1111/pme.12365.

6. Heiberg J, Hansen L, Wemmelund K, et al. Point-of-Care Clinical Ultrasound for Medical Students. Ultrasound Int Open. 2015;01(02):E58-E66. doi:10.1055/s0035-1565173.

7. Barrington MJ, Viero LP, Kluger R, Clarke AL, Ivanusic JJ, Wong DM. Determining the Learning Curve for Acquiring Core Sonographic Skills for 33 Ultrasound-Guided Axillary Brachial Plexus Block. Reg Anesth Pain Med. 2016;41(6):667-670. doi:10.1097/AAP.0000000000000487.

8. Vitto MJ, Myers M, Vitto CM, Evans DP. Perceived Difficulty and Success Rate of Standard Versus Ultrasound-Guided Peripheral Intravenous Cannulation in a Novice Study Group. J Ultrasound Med. 2016;35(5):895-898. doi:10.7863/ultra.15.06057.

9. Hoyer R, Means R, Robertson J, et al. Ultrasound-guided procedures in medical education: a fresh look at cadavers. Intern Emerg Med. 2016;11(3):431-436. doi:10.1007/s11739-015-1292-7.

10. Amini R, Stolz LA, Breshears E, et al. Assessment of ultrasound-guided procedures in preclinical years. Intern Emerg Med. 2016;12(7):1-7. doi:10.1007/s11739-016-1525-4.

**Domain 3. Principles of an International Consensus Curriculum**

**3.1 The ultrasound curriculum forms the foundation for ultrasound training along a continuum of medical education from undergraduate through graduate to continuing medical education***.*

Point of Care Ultrasound (POCUS) at the patient's bedside represents a new tool for the practicing physician. Originally introduced by Emergency Medicine as a means to rapidly evaluate critical patients in the emergency room, its use has spread to intensive care units, inpatient services, and outpatient care settings. Already, as many as 20 medical and surgical specialties now require competency and/or experience in ultrasound applications at the completion of their graduate medical education training. (1) Because POCUS is rapidly diffusing into medical practice, it is important that there be a structured and well-organized program to facilitate training in schools of medicine, and also be applicable to all providers as initial exposure to POCUS learning and practice.

Many medical schools have integrated some aspect of ultrasound into their education programs, often into the pre-clinical science years, as a means of enhancing clinicopathological understanding. (2-5) Additionally, over the last decade a number of US medical US schools have integrated ultrasound into their four-year curriculum, demonstrating its feasibility and initial impact on its students. (6-8). A recent scoping review of the burgeoning literature on ultrasound in medical school education supports the need for a standardized point-of-care ultrasound curriculum that would lead to the development of common standards for milestones and competency-based assessments. (9, 10) Hence, a foundational curriculum delineated by experts in the field of ultrasonography, by those experienced in its use in diverse clinical settings and at the point of patient care, and by educators knowledgeable about the trajectory of physician development can provide guidance as this new skill is integrated into the profession throughout the world. This international consensus curriculum delineates the foundational knowledge, skills, and attitudes that are necessary for the acquisition of competence in POCUS throughout professional development.

**References**

1. “ACGME Home.” Accessed December 19, 2018.

2. Bahner, David P., Ellen Goldman, David Way, Nelson A. Royall, and Yiju Teresa Liu. “The State of Ultrasound Education in U.S. Medical Schools: Results of a National Survey.” Academic Medicine: Journal of the Association of American Medical Colleges 89, no. 12 (December n2014)

3. Dinh, Vi Am, Jasmine Y. Fu, Samantha Lu, Alan Chiem, J. Christian Fox, and Michael Blaivas. “Integration of Ultrasound in Medical Education at United States Medical Schools.” Journal of Ultrasound in Medicine 35, no. 2 (2016): 413–19.

4. Afonso, Nelia, David Amponsah, James Yang, Jennifer Mendez, Patrick Bridge, Gregory Hays, Sudhir Baliga, et al. “Adding New Tools to the Black Bag—Introduction of Ultrasound into the Physical Diagnosis Course.” Journal of General Internal Medicine 25, no. 11 (November 2010): 1248–52.

5. Dinh, Vi Am, Jon Frederick, Rebekah Bartos, Tamara M. Shankel, and Leonard Werner. “Effects of Ultrasound Implementation on Physical Examination Learning and Teaching During the First Year of Medical Education.” Journal of Ultrasound in Medicine 34, no. 1 (2015): 43–50.

6. Hoppmann, Richard A., Victor V. Rao, Floyd Bell, Mary Beth Poston, Duncan B. Howe, Shaun Riffle, Stephen Harris, et al. “The Evolution of an Integrated Ultrasound Curriculum (IUSC) for Medical Students: 9-Year Experience.” Critical Ultrasound Journal 7 (November 21, 2015).

7. Bahner, David P., Eric J. Adkins, Daralee Hughes, Michael Barrie, Creagh T. Boulger, and Nelson A. Royall. “Integrated Medical School Ultrasound: Development of an Ultrasound Vertical Curriculum.” Critical Ultrasound Journal 5, no. 1 (July 2, 2013): 6.

8. Wilson, Sean P., Jason M. Mefford, Shadi Lahham, Shahram Lotfipour, Mohammad Subeh, Gracie Maldonado, Sophie Spann, and John C. Fox. “Implementation of a 4-Year Point-of-Care Ultrasound Curriculum in a Liaison Committee on Medical Education–Accredited US Medical School.” Journal of Ultrasound in Medicine 36, no. 2 (2017): 321–25. 36

9. Tarique, Usman, Brandon Tang, Manni Singh, Kulamakan Mahan Kulasegaram, and Jonathan Ailon. “Ultrasound Curricula in Undergraduate Medical Education: A Scoping Review.” Journal of Ultrasound in Medicine 37, no. 1 (2018): 69–82.

10. Dinh, Vi Am, Daniel Lakoff, Jamie Hess, David P. Bahner, Richard Hoppmann, Michael Blaivas, John S. Pellerito, Alfred Abuhamad, and Sorabh Khandelwal. “Medical Student Core Clinical Ultrasound Milestones: A Consensus Among Directors in the United States.” Journal of Ultrasound in Medicine 35, no. 2 (February 2016): 421–34.

**3.2 The ultrasound curriculum supports undergraduate medical education.**

**3.3 The ultrasound curriculum prepares learners for future additional clinical training and/or practice opportunities.**

Ultrasound curricula have been integrated into pre-clinical studies and the early clinical knowledge, skills, and attitudes required for all pre-doctoral students. (2-6) As such, the learner develops a foundation for the understanding and utilization of ultrasound that is applicable to future clinical practice, including residency training programs that use ultrasound in their specialty areas. (1,7) Pratt Bahner et al surveyed their five-year experience with students who had participated in a senior year (fourth) longitudinal elective in ultrasound and compared the results to those who had not taken this year long elective. They found that participants in the elective were more likely to seek additional training in their post-graduate residency programs, to evaluate themselves as more proficient in POCUS, and ultimately to use ultrasound in their clinical practice. (8).

**References**

1. “ACGME Home.” Accessed December 19, 2018.

2. Bahner, David P., Ellen Goldman, David Way, Nelson A. Royall, and Yiju Teresa Liu. “The State of Ultrasound Education in U.S. Medical Schools: Results of a National Survey.” Academic Medicine: Journal of the Association of American Medical Colleges 89, no. 12 (December n2014)

3. Dinh, Vi Am, Jasmine Y. Fu, Samantha Lu, Alan Chiem, J. Christian Fox, and Michael Blaivas. “Integration of Ultrasound in Medical Education at United States Medical Schools.” Journal of Ultrasound in Medicine 35, no. 2 (2016): 413–19. 38

4. Afonso, Nelia, David Amponsah, James Yang, Jennifer Mendez, Patrick Bridge, Gregory Hays, Sudhir Baliga, et al. “Adding New Tools to the Black Bag—Introduction of Ultrasound into the Physical Diagnosis Course.” Journal of General Internal Medicine 25, no. 11 (November 2010): 1248–52.

5. Dinh, Vi Am, Jon Frederick, Rebekah Bartos, Tamara M. Shankel, and Leonard Werner. “Effects of Ultrasound Implementation on Physical Examination Learning and Teaching During the First Year of Medical Education.” Journal of Ultrasound in Medicine 34, no. 1 (2015): 43–50.

6. Tarique, Usman, Brandon Tang, Manni Singh, Kulamakan Mahan Kulasegaram, and Jonathan Ailon. “Ultrasound Curricula in Undergraduate Medical Education: A Scoping Review.” Journal of Ultrasound in Medicine 37, no. 1 (2018): 69–82.

7. Heinzow, Hauke S, Hendrik Friederichs, Philipp Lenz, Andre Schmedt, Jan C Becker, Karin Hengst, Bernhard Marschall, and Dirk Domagk. “Teaching Ultrasound in a Curricular Course According to Certified EFSUMB Standards during Undergraduate Medical Education: A Prospective Study.” BMC Medical Education 13 (June 11, 2013): 84.

8. Prats, Michael I., Nelson A. Royall, Ashish R. Panchal, David P. Way, and David P. Bahner. “Outcomes of an Advanced Ultrasound Elective.” Journal of Ultrasound in Medicine 35, no. 5 (2016): 975–82.

**3.4 The ultrasound curriculum is developed in accordance with accepted standards for medical education as defined by national and international accrediting bodies.**

Programs throughout the world that lead to the doctor of medicine degree (or its equivalent) are accredited by bodies that set standards for these educational programs. Ultrasound curricula, which are increasingly included in the course of study, can support and enhance compliance with standards that are required for the undergraduate medical education program in medicine. (1-6) As an example, the LCME, which sets standards for all US and Canadian allopathic medical schools, has 12 standards. The ultrasound curriculum, as implemented in the MD educational program, supports the following standards of the LCME (2): an outcomes-based curriculum where learner progress of competence would be measurable (element 6.1); it supports a breadth and depth of education for all students that will allow them to practice in contemporary medical environments (standard 7); because the use of ultrasound crosses many fields of expertise, it lends itself to interprofessional collaboration (element 7.9); and, the successful integration and coordination of an ultrasound curriculum must fall to oversight by the institution (element 8.1).

**References**

1. Heinzow, Hauke S, Hendrik Friederichs, Philipp Lenz, Andre Schmedt, Jan C Becker, Karin Hengst, Bernhard Marschall, and Dirk Domagk. “Teaching Ultrasound in a Curricular Course According to Certified EFSUMB Standards during Undergraduate Medical Education: A Prospective Study.” BMC Medical Education 13 (June 11, 2013): 84.

2. Liaison Committee on Medical Education (LCME): “Standards, Publications, & Notification Forms | LCME.” Accessed December 28, 2018.

3. Steinmetz, Peter, Octavian Dobrescu, Sharon Oleskevich, and John Lewis. “Bedside Ultrasound Education in Canadian Medical Schools: A National 40 Survey.” Canadian Medical Education Journal 7, no. 1 (March 31, 2016): e78– 86.

4. Russ, Brian A., Danika Evans, Daniel Morrad, Codee Champney, Ashley M. Woodworth, Lane Thaut, and Molly Thiessen. “Integrating Point-of-Care Ultrasonography Into the Osteopathic Medical School Curriculum.” The Journal of the American Osteopathic Association 117, no. 7 (July 1, 2017): 451–56.

5. Wakefield, Richard J., Asoka Weerasinghe, Patrick Tung, Laura Smith, James Pickering, Tendekayi Msimanga, Mohit Arora, et al. “The Development of a Pragmatic, Clinically Driven Ultrasound Curriculum in a UK Medical School.” Medical Teacher 40, no. 6 (June 3, 2018): 600–606.

6. Baltarowich, Oksana H., Donald N. Di Salvo, Leslie M. Scoutt, Douglas L. Brown, Christian W. Cox, Michael A. DiPietro, Daniel I. Glazer, et al. “National Ultrasound Curriculum for Medical Students.” Ultrasound Quarterly 30, no. 1 (March 2014): 13–19.

**3.5 The ultrasound curriculum lends itself to a competency-based model that includes measurable outcomes and markers of progression toward those outcomes (milestones).**

**3.6 The ultrasound curriculum can incorporate ultrasound knowledge, skills, attitudes, and professional judgment into entrustable professional activities (EPAs) as appropriate for patient care.**

The ultrasound curriculum follows current educational standards that acknowledge the importance of clear measurable markers of knowledge and skill acquisition for the learner and the program. This curriculum facilitates ultrasound integration into the medical school curriculum and can be translated into milestones with measurable markers. (1-3). Dinh et al, using a modified Delphi technique, was able to arrive at consensus on 204 milestones, as supported by 34 program directors in medical school ultrasound education in the United States. (2)

Entrustable Professional Activities (EPAs), as tasks or responsibilities that students or trainees are competent to undertake without supervision, likewise can readily integrate ultrasound. Of the 13 EPAs considered for transition from medical school to post-graduate training, POCUS can enhance competence in at least seven of the listed 13 desired competencies. (4)* Likewise, of the five EPAs considered for transition of students from pre-clinical to clinical work, POCUS can readily enhance three of these: a) gathering of patient information; b) the integration of that information and c) information sharing with patients. (5)

*13 EPAs: Gather a history and perform a physical examination, Prioritize a differential diagnosis following a clinical encounter, Recommend and interpret common diagnostic and screening tests, Enter and discuss orders/prescriptions, Document a clinical encounter in the patient record, Provide an oral presentation of a clinical encounter, Form clinical questions and retrieve evidence to advance patient care, Give or receive a patient handover to transition care responsibility, Collaborate as a member of an interprofessional team, Recognize a patient requiring urgent or emergent care and initiate evaluation and management, Obtain informed consent for tests and/or procedures, Perform general procedures of a physician); identify system failures and contribute to a culture of safety and improvement.

**References**

1. Wilson, Sean P., Jason M. Mefford, Shadi Lahham, Shahram Lotfipour, Mohammad Subeh, Gracie Maldonado, Sophie Spann, and John C. Fox. “Implementation of a 4-Year Point-of-Care Ultrasound Curriculum in a Liaison Committee on Medical Education–Accredited US Medical School.” Journal of Ultrasound in Medicine 36, no. 2 (2017): 321–25.

2. Dinh, Vi Am, Daniel Lakoff, Jamie Hess, David P. Bahner, Richard Hoppmann, Michael Blaivas, John S. Pellerito, Alfred Abuhamad, and Sorabh Khandelwal. “Medical Student Core Clinical Ultrasound Milestones: A Consensus Among Directors in the United States.” Journal of Ultrasound in Medicine 35, no. 2 (February 2016): 421–34.

3. Serrao, Graziano, Massimo Tassoni, Alberto M. Magenta-Biasina, Antonio G. Mantero, Antonino M. Previtera, Michela C. Turci, Elia M. Biganzoli, and Emanuela M. Bertolini. “Competency-Based Medical Education Studying Live Anatomy by Ultrasound.” International Journal of Medical Education 8 (July 19, 2017): 268–69.

4. Lomis, Kimberly, Jonathan M. Amiel, Michael S. Ryan, Karin Esposito, Michael Green, Alex Stagnaro-Green, Janet Bull, George C. Mejicano, and for the AAMC Core EPAs for Entering Residency Pilot Team. “Implementing an Entrustable Professional Activities Framework in Undergraduate Medical Education: Early Lessons From the AAMC Core Entrustable Professional Activities for Entering Residency Pilot.” Academic Medicine 92, no. 6 (June 2017).

5. Chen, H. Carrie, Margaret McNamara, Arianne Teherani, Olle ten Cate, and Patricia O’Sullivan. “Developing Entrustable Professional Activities for Entry into Clerkship.” Academic Medicine 91, no. 2 (February 2016): 247

**3.7 The ultrasound curriculum enhances the learning of basic sciences that are relevant to the understanding of human pathophysiology and the practice of medicine.**

Ultrasound serves as an adjunct to visualize normal and abnormal human structure and function. (1-4) Integrating ultrasound into the teaching of pre-clinical sciences can improve understanding and enhance learning outcomes that are important for future clinical practice. (4-6) When surveyed, students see this as a valuable tool to enhance their learning in these areas. (6)

**References**

1. Tarique, Usman, Brandon Tang, Manni Singh, Kulamakan Mahan Kulasegaram, and Jonathan Ailon. “Ultrasound Curricula in Undergraduate Medical Education: A Scoping Review.” Journal of Ultrasound in Medicine 37, no. 1 (2018): 69–82.

2. Baltarowich, Oksana H., Donald N. Di Salvo, Leslie M. Scoutt, Douglas L. Brown, Christian W. Cox, Michael A. DiPietro, Daniel I. Glazer, et al. “National Ultrasound Curriculum for Medical Students.” Ultrasound Quarterly 30, no. 1 (March 2014): 13–19.

3. Patel, Shilpan G., Brion Benninger, and S. Ali Mirjalili. “Integrating Ultrasound into Modern Medical Curricula.” Clinical Anatomy (New York, N.Y.) 30, no. 4 (May 2017): 452–60.

4. Bell, Floyd E., L. Britt Wilson, and Richard A. Hoppmann. “Using Ultrasound to Teach Medical Students Cardiac Physiology.” Advances in Physiology Education 39, no. 4 (December 2015): 392–96.

5. Hammoudi, Nadjib, Dimitri Arangalage, Lila Boubrit, Marie Christine Renaud, Richard Isnard, Jean-Philippe Collet, Ariel Cohen, and Alexandre 45 Duguet. “Ultrasound-Based Teaching of Cardiac Anatomy and Physiology to Undergraduate Medical Students.” Archives of Cardiovascular Diseases 106, no. 10 (October 1, 2013): 487–91.

6. So, Sokpoleak, Rita M. Patel, and Steven L. Orebaugh. “Ultrasound Imaging in Medical Student Education: Impact on Learning Anatomy and Physical Diagnosis.” Anatomical Sciences Education 10, no. 2 (2017): 176–89.

**3.8 The ultrasound curriculum enhances the learning of clinical sciences through the integration of ultrasound into clinical problem solving.**

**3.9 The ultrasound curriculum enhances the learning of clinical sciences through the care of patients at their point of care**.

Along with the integration of patient history, the standard physical exam, and basic laboratory data, point of care ultrasound can provide additional information readily available at the time of the patient encounter in order to more rapidly and accurately guide diagnosis and treatment. (1-2). Thus, the introduction of ultrasound into the medical school curriculum, likewise, may provide additional accuracy in the accumulation of patient information that fosters improved understanding of underlying pathophysiology to arrive at a rational diagnostic or therapeutic plan. Ultrasound in undergraduate medical education has been shown to improve the accuracy of the student physical examination. For example, students with limited ultrasound training were more accurate than cardiologists in cardiac exams (3); than faculty in estimating the size of the liver (4); and in locating the femoral artery with than without ultrasound. (5) Integration of ultrasound has the potential to improve other aspects of the physical exam, including evidence of professionalism. 6) Use of ultrasound by students may enhance their ability to assess patients with critical presentations, such as hypotension. (7) Accurate patient assessment during physical examination allows the student to better integrate findings into their overall clinical problem solving.

**References**

1. Alpert, Joseph S., Jeanette Mladenovic, and David B. Hellmann. “Should a Hand-Carried Ultrasound Machine Become Standard Equipment for Every Internist?” The American Journal of Medicine 122, no. 1 (January 2009): 1–3. 47

2. Moore, Christopher L., and Joshua A. Copel. “Point-of-Care Ultrasonography.” New England Journal of Medicine 364, no. 8 (February 24, 2011): 749–57.

3. Kobal, Sergio L., Luca Trento, Simin Baharami, Kirsten Tolstrup, Tasneem Z. Naqvi, Bojan Cercek, Yoram Neuman, et al. “Comparison of Effectiveness of Hand-Carried Ultrasound to Bedside Cardiovascular Physical Examination.”American Journal of Cardiology 96, no.7 (October 1, 2005):1002–6.

4. Mouratev, Gueorgui, Duncan Howe, Richard Hoppmann, Mary Beth Poston, Rodney Reid, James Varnadoe, Stuart Smith, Brown McCallum, Victor Rao, and Paul DeMarco. “Teaching Medical Students Ultrasound to Measure Liver Size: Comparison with Experienced Clinicians Using Physical Examination Alone.” Teaching and Learning in Medicine 25, no. 1 (2013): 84–88.

5. Ahn, Justin S., Andrew J. French, Molly E. W. Thiessen, Vaughn Browne, Mark Deutchman, Gretchen Guiton, Wendy Madigosky, and John L. Kendall. “Using Ultrasound to Enhance Medical Students’ Femoral Vascular Physical Examination Skills.” Journal of Ultrasound in Medicine 34, no. 10 (2015): 1771– 76.

6. Dinh, Vi Am, Jon Frederick, Rebekah Bartos, Tamara M. Shankel, and Leonard Werner. “Effects of Ultrasound Implementation on Physical Examination Learning and Teaching During the First Year of Medical Education.” Journal of Ultrasound in Medicine 34, no. 1 (2015): 43–50.

7. Amini, Richard, Lori A Stolz, Nicholas C Hernandez, Kevin Gaskin, Nicola Baker, Arthur Barry Sanders, and Srikar Adhikari. “Sonography and Hypotension: A Change to Critical Problem Solving in Undergraduate Medical Education.” Advances in Medical Education and Practice 7 (January 14, 2016): \

**3.10 The ultrasound curriculum includes opportunities for self-directed learning and assessment.**

**3.11 The ultrasound curriculum encourages life-long learning.**

The calls for reform in medical education emphasize the need for individualized learning with a range of opportunities to support varying rates of skill development and competency attainment, while also laying the groundwork for ongoing professional development. (3) The numerous examples of ultrasound curricula to date have demonstrated the variability in approaches that support the acquisition of the skills, attitudes, and knowledge that are necessary to gain competence. (1) All require student directed learning and practice for success. The foundational curriculum answers a call for a standardized curriculum that allows students and all practitioners to reach milestones at an individual pace. (1-2) It underscores the ongoing acquisition of ultrasound expertise and development of skills over time and as tailored for individual practice.

**References**

1. Tarique, Usman, Brandon Tang, Manni Singh, Kulamakan Mahan Kulasegaram, and Jonathan Ailon. “Ultrasound Curricula in Undergraduate Medical Education: A Scoping Review.” Journal of Ultrasound in Medicine 37, no. 1 (2018): 69–82.

2. Dinh, Vi Am, Daniel Lakoff, Jamie Hess, David P. Bahner, Richard Hoppmann, Michael Blaivas, John S. Pellerito, Alfred Abuhamad, and Sorabh Khandelwal. “Medical Student Core Clinical Ultrasound Milestones: A Consensus Among Directors in the United States.” Journal of Ultrasound in Medicine 35, no. 2 (February 2016): 421–34. 49

3. Irby, David M., Molly Cooke, and Bridget C. OʼBrien. “Calls for Reform of Medical Education by the Carnegie Foundation for the Advancement of Teaching: 1910 and 2010:” Academic Medicine 85, no. 2 (February 2010): 220–27.

**3.12 The ultrasound curriculum is based on evidence and expert opinion.**

**3.13 The ultrasound curriculum is consistent with recommendations and guidelines of well-**

**established specialty organizations.**

**3.14 The ultrasound curriculum is consistent with recommendations and guidelines of regulatory**

**bodies with significant experience in ultrasound.**

Point of care Ultrasound (POCUS) represents a new clinical skill, with much information now accumulating on its applicability to many areas of medicine. As such, a burgeoning literature along with expert opinion is becoming widely accessible to guide the development of an international curriculum. Several societies have developed guidelines and/or curricula in the area of ultrasound (1-9), and others are in the process of developing these guidelines (10-11). The International Consensus Curriculum aligns with these society guidelines to prepare early learners with the necessary foundation to use POCUS in their future chosen area of medicine, as supported by the guidelines of these national and international societies.

**References**

1. Heinzow, Hauke S, Hendrik Friederichs, Philipp Lenz, Andre Schmedt, Jan C Becker, Karin

Hengst, Bernhard Marschall, and Dirk Domagk. “Teaching Ultrasound in a Curricular Course According to Certified EFSUMB Standards 51 during Undergraduate Medical Education: A Prospective Study.” BMC Medical Education 13 (June 11, 2013): 84.

2. America Institute for Ultrasound and Medicine

3. American College of Emergency Physicians

4. American Society of Cardiac Echocardiography

5. Society for Critical Care Medicine

6. Via, Gabriele, Arif Hussain, Mike Wells, Robert Reardon, Mahmoud ElBarbary, Vicki E. Noble, James W. Tsung, et al. “International EvidenceBased Recommendations for Focused Cardiac Ultrasound.” Journal of the American Society of Echocardiography: Official Publication of the American Society of Echocardiography 27, no. 7 (July 2014): 683.e1-683.e33.

7. International Liaison Committee on Lung Ultrasound (ILC-LUS) for the International Consensus Conference on Lung Ultrasound (ICC-LUS), Giovanni Volpicelli, Mahmoud Elbarbary, Michael Blaivas, Daniel A. Lichtenstein, Gebhard Mathis, Andrew W. Kirkpatrick, et al. “International 39. EvidenceBased Recommendations for Point-of-Care Lung Ultrasound.” Intensive Care Medicine 38, no. 4 (April 2012): 577–91.

8. Dancel, Ria, Daniel Schnobrich, Nitin Puri, Ricardo Franco-Sadud, Joel Cho, Loretta Grikis, Brian P. Lucas, Mahmoud El-Barbary, Society of Hospital Medicine Point of Care Ultrasound Task Force, and Nilam J. Soni. “Recommendations on the Use of Ultrasound Guidance for Adult Thoracentesis: A Position Statement of the Society of Hospital Medicine.” Journal of Hospital Medicine 13, no. 2 (2018): 126–35.

9. American Academy of Family Physicians Recommended Curriculum Guidelines.

10. Society for Hospital Medicine

11. American College of Physicians
