# Supplementary material for: International consensus conference recommendations on ultrasound education for undergraduate medical students
Source: Ultrasound J. 2022 Jul 27;14:31. doi: 10.1186/s13089-022-00279-1 (PMC9329507; doi:10.1186/s13089-022-00279-1)
Supplement: Supplementary file 2 — Additional file 2: Appendix S2. Recommended medical student ultrasound curricular content. [file 13089_2022_279_MOESM2_ESM.docx]

**Appendix 2**

**Recommended Medical Student Ultrasound Curricular Content**

***Indicates Strong Recommendation**

| **Part I: Basic Foundations of POCUS** | | | |
| --- | --- | --- | --- |
| **Be familiar with terms:** | | **Explain fundamental principles:** | **Demonstrate an understanding of**  **probe components and parts:** |
| Wavelength* | Scatter* | B mode* | Housing/body* |
| Amplitude* | Transmission* | M Mode* | Piezoelectric crystals* |
| Frequency* | Resolution* | Color Flow* | Marker/indicator* |
| Attenuation* | Reflection* | Power Doppler | Cord* |
| Refraction* | Aliasing | Spectral Doppler |  |
| Absorption* |  |  |  |

| **Know indications/limitations of probes:** | **Demonstrate Appropriate:** | **Utilize transducer manipulations:** |
| --- | --- | --- |
| Linear* | Probe storage* | Slide* |
| Curved array* | Probe care* | Rock* |
| Phased array* | Probe Cleaning/Disinfection* | Sweep |
| Endocavity |  | Fan |
|  |  | Pressure/compression |
|  |  | Rotation |

| **Be familiar with image descriptions:** | **Be able to discuss sonographic**  **characteristics of tissues:** | **Demonstrate ability to optimize**  **Image with machine adjustments:** |
| --- | --- | --- |
| In-plane and out-of-plane* | Anechoic* | Presets* |
| Deep and superficial* | Hyperechoic* | Gain* |
| Medial and lateral | Hypoechoic | Time Gain Compensation |
| Cranial and caudal | Isoechoic | Frequency |
| Coronal* | Mixed echogenicity* | Depth* |
| Sagittal* | Homogeneous* | Focal point |
| Transverse* | Heterogeneous* | Probe marker* |
|  | Solid* |  |
|  | Cystic* |  |

| **Recognize ultrasound artifacts and explain the cause:** | | **Describe indications for:** | **Acquire images with:** |
| --- | --- | --- | --- |
| Reverberation (A and B lines)* | Acoustic enhancement* | Brightness B mode* | Brightness B mode* |
| Comet tail | Acoustic shadowing* | Motion M mode* | Motion M mode* |
| Posterior acoustic shadowing* | Mirror image* | Doppler flow* | Color Doppler |
| Air artifact* | Twinkle | Power Doppler |  |
| Mirroring* |  | Spectral Doppler |  |

| **Identify tissues:** | **Demonstrate proper patient care:** |
| --- | --- |
| Fluid* | Professional communication of ultrasound use * |
| Fat | Obtaining informed consent* |
| Soft tissue* | Respect for patient privacy* |
| Bone* | Respect for patient comfort* |
| Muscle | Appropriate positioning of the patient* |
| Cartilage | Completion of documentation of findings* |
| Tendon | An understanding of the principle of ALARA  (As Low As Reasonably Achievable)* |
| Nerve |  |
| Blood Vessels |  |
| **Correlate ultrasound images with clinical findings*** | |

| **Part II: Specific views, structures, and pathology** | | |
| --- | --- | --- |
| **Heart and Vessels** | | |
|  | | |
| **Views:** | **Structures and physiology:** | **Clinical Pathology**: |
| Parasternal Long Axis* | Left atrium, right atrium* | Poor contractility* |
| Parasternal Short Axis | Left ventricle, right ventricle* | LVEF less than 40% |
| Apical Four Chamber | Mitral valve* | LVEF greater than 40% |
| Subxiphoid (subcostal)* | Aortic valve* | Enlarged chamber size* |
| IVC Transverse | Tricuspid valve | Enlarged Left atrium |
| IVC Longitudinal | Myocardium* | Enlarged Left ventricle |
|  | Pericardium* | Enlarged Right Ventricle |
|  | Descending Aorta | Distinguish arterial/venous Doppler flow |
|  | Aortic arch | Presence of pericardial effusion* |
|  | Abdominal aorta* | Distinguish pleural from pericardial effusion |
|  | Aortic bifurcation - iliac arteries | Left Ventricular Hypertrophy |
|  | Correlation sonographic cardiac cycle with EKG | Right ventricular strain from PE |
|  | Carotid arteries, including common carotid* | Size of abdominal aortic aneurysm |
|  | Inferior Vena Cava* | Decreased volume by IVC collapsibility |
|  | IVC size* | Lower extremity deep venous thrombosis |
|  | IVC respiratory variations* |  |
|  | Internal jugular vein* |  |

| **Lungs and Chest** | | |
| --- | --- | --- |
|  | | |
| **Views:** | **Structures and physiology:** | **Clinical Pathology:** |
| Anterior chest bilaterally* | Visceral pleura | B lines - B Profile* |
| Lateral and posterior chest* | Parietal pleura | Pneumothorax - No pleural sliding |
| Longitudinal across two ribs* | Lung sliding* | Lung Point |
| Costophrenic angles bilaterally* | A lines - A Profile* | Pulmonary edema |
|  |  | Pleural effusion* |
|  |  | Presence of consolidation |
|  |  | Sliding Curtain Sign |

Acute Respiratory Distress Syndrome

| **Abdomen** | | | |
| --- | --- | --- | --- |
|  | | | |
| **Views:** | **Structures and physiology:** | | **Pathology:** |
| Epigastric* | Liver* | Pelvis | Ascites* |
| Left upper quadrant* | Size | Calyces | Hemoperitoneum* |
| Right upper quadrant* | Parenchyma | Spleen* | Hydronephrosis* |
| Lower abdomen* | Portal vein | Small bowel | Sonographic Murphy Sign |
|  | Hepatic vein | Peristalsis | Cholelithiasis* |
|  | Gallbladder* | Right and left costophrenic angles* |  |
|  | Stomach | Subdiaphragmatic space |  |
|  | Right/ left kidneys* | Hepatorenal space (Morison’s pouch)* |  |
|  | Size | Peri-splenic area for fluid* |  |
|  | Cortex | Splenorenal |  |
|  |  |  |  |
|  |  |  |  |

| **Pelvis** | | |
| --- | --- | --- |
|  | | |
| **Views:** | **Structures** | **Pathology** |
| Urinary Bladder, longitudinal* | Bladder, volume | Free fluid* |
| Urinary Bladder, transverse* | Uterus* | Distended bladder* |
| Uterus, transabdominal, long* | Fetal number | Foley catheter position |
| Uterus, transabdominal, trans* | Fetal heartbeat |  |
|  | Fetal position |  |

| **Head and Neck** | | |
| --- | --- | --- |
|  | | |
| **Views:** | **Structures** | **Pathology** |
| Longitudinal* | Muscles of the neck | Thyromegaly |
| Transverse* | Thyroid lobes | Thyroid mass or cysts |
|  | Thyroid isthmus | Presence of endotracheal tube |
|  | Trachea | Esophageal intubation |
|  | Esophagus | Eye globe |
|  | Globe of the eye |  |
|  | Optic nerve |  |

| **Musculoskeletal** | | | | |
| --- | --- | --- | --- | --- |
|  | | | | |
| **Views:** | | **Views of specific Joints:** | | **Structures in General:** |
| Views in general* | | Elbow, long | | Dermis & SC Tissues |
| Transverse* | | Elbow, trans | | Tendons |
| Longitudinal* | | Wrist, long | | Ligaments |
|  | | Wrist, trans | | Cortex of bone |
|  | | Knee, long | | Joint space |
|  | | Knee, trans | | Fat pads |
|  | |  | | Synovium |
|  | | | | |
| **Specific joint structures:** | | | **Pathology:** | |
| Distal radius | Achilles tendon | | Joint effusions | |
| Distal ulna | Distal fibula | | Bursal fluid | |
| Quadriceps tendon | Distal tibia | | Soft tissue edema/cobblestoning | |
| Bursa, suprapatellar | Shoulder Humeral Head | | Soft tissue abscess or cyst | |
| Patella | Shoulder Glenoid | | Soft tissue solid mass | |
| Patellar tendon | Shoulder Clavicle | |  | |
| Tibial tuberosity | Shoulder Biceps tendon | |  | |

| **Part III: Procedures / Protocols** | |
| --- | --- |
| **Procedures:** | **Protocols:** |
| Peripheral Vein Cannulation (PIV) | E-FAST protocol |
| Central Venous Cannulation (CVC) | RUSH protocol |
| Paracentesis |  |
| Thoracentesis |  |
| Arthrocentesis |  |
| Visualize fluid-filled cavities* |  |
| Demonstrate guiding a needle into fluid-filled  cavity, on patients or a phantom model* |  |
